# A novel two-phase robust portfolio selection and optimization approach under uncertainty: A case study of Tehran stock exchange

**DOI:** 10.1371/journal.pone.0239810

**Published:** 2020-10-12

**Authors:** Pejman Peykani, Emran Mohammadi, Armin Jabbarzadeh, Mohsen Rostamy-Malkhalifeh, Mir Saman Pishvaee

**Affiliations:** 1 School of Industrial Engineering, Iran University of Science and Technology, Tehran, Iran; 2 Department of Mathematics, Faculty of Science, Science and Research Branch, Islamic Azad University, Tehran, Iran; The Bucharest University of Economic Studies, ROMANIA

## Abstract

Portfolio construction is one of the most critical problems in financial markets. In this paper, a new two-phase robust portfolio selection and optimization approach is proposed to deal with the uncertainty of the data, increasing the robustness of investment process against uncertainty, decreasing computational complexity, and comprehensive assessments of stocks from different financial aspects and criteria are provided. In the first phase of this approach, all candidate stocks’ efficiency is measured using a robust data envelopment analysis (RDEA) method. Then in the second phase, by applying robust mean-semi variance-liquidity (RMSVL) and robust mean-absolute deviation-liquidity (RMADL) models, the amount of investment in each qualified stock is determined. Finally, the proposed approach is implemented in a real case study of the Tehran stock exchange (TSE). Additionally, a sensitivity analysis of all robust models of this study is examined. Illustrative results show that the proposed approach is effective for portfolio selection and optimization in the presence of uncertain data.

## 1. Introduction

The portfolio selection and optimization problems are two of the main branches of studies in investment management. Extensive researches have been done on the portfolio selection problem from different viewpoints [1–3]. The most important research in this area has been by Markowitz [4]. He presented the concept of diversity in the portfolio selection problem. In the original Markowitz’s [4] model, the portfolio selection problem is developed by only two criteria, i.e., risk and return. However, the decision to purchase a stock and select a portfolio of stocks can be more difficult since many attributes must be considered simultaneously. Some of these attributes may include the rate of return, the rate of liquidity, systematic risk, non-systematic risk, financial ratios, etc. Decision-makers (DMs) and investors can use the multi-criteria decision making (MCDM) approach to consider more than two criteria in selecting stocks [5].

Data envelopment analysis (DEA) is one of the popular and powerful MCDM approaches applied to reach this goal. DEA estimates the relative efficiency of decision-making units (DMUs) considering the multiple inputs and multiple outputs [6–8]. DEA can be implemented in portfolio construction by measuring stocks’ efficiency to recognize good stocks and filter bad stocks. It should be noted that in classic DEA models, each DMU could specify a set of weights that show it in the most favorable condition in comparison to other DMUs. This flexibility in choosing weights for each DMU caused that the efficiency of stocks to be considered optimistic. Thus, to propose the conservative approach and resolve this issue, after filtering the undesirable stocks and detecting the most desirable stocks, it is needed to reevaluate the qualified stocks in another phase in order to assign the amount of investment in each stock.

Another point that should be considered in the proposed approach for portfolio construction is the uncertain nature of parameters [9–12]. Because in the real-world, we face uncertain data, and one of the most important features of financial markets is their embedded uncertainty. Also, one of the most important assumptions in DEA is that the measured data are certain. However, a little bias or deviation in data’s values can cause significant differences in the results. In a worst-case, we will face infeasible solutions. Especially when the efficiencies of units are close, it is essential to develop a procedure and models for ranking the stocks and, consequently, decision-making about weights of the stocks in the portfolio that is capable of being employed under uncertainty. Robust optimization (RO) methodology is one of the popular methods that can be used to deal with uncertainty [13–15].

The goal of the current study is to propose a robust two-phase approach for portfolio construction problem by using data envelopment analysis and robust optimization approaches. In the first phase, the efficiency of all stocks that can be invested, are evaluated and measured. At the end of this phase, only the stocks that pass the filter of the investor are qualified for a candidate to be invested in the second phase. In this phase, DEA models are used. Then, in the second phase, the amount invested in each qualified stock is decided, and finally, the portfolio will be created. In this phase, mean-semi variance-liquidity (MSVL) and mean-absolute deviation-liquidity (MADL) models are used. It should be noted that in each phase, uncertainty is considered by a robust optimization method. Finally, the proposed approach of paper will be implemented in a real case study of the Tehran stock exchange (TSE).

The main advantages of the proposed approach in this study can be summarized as follows: (1) the presented approach can be applied in the presence of uncertain data, (2) computational complexity of portfolio optimization is decreased by the first phase in order to satisfy cardinality constraint, (3) conservatism levels of the investment process is increased using of two-phases method and considering uncertainty, (4) all candidate stocks for investment are comprehensively assessed from different financial aspects and criteria by employing the MCDM approaches.

The rest of this paper is organized as follows. The literature and research gaps are reviewed in Section 2. The nomenclatures and background of the paper is explained in Section 3, which contains the classic portfolio models, basic DEA models, and main robust optimization approaches. Two phases approach for portfolio construction problem of this research is presented in Section 4. The proposed approach for the portfolio selection problem is implemented for a real case study of the Tehran stock exchange that will be presented in Section 5. All of the proposed models have been studied using sensitivity analysis in Section 6. Finally, the conclusions of this study and some directions for future research are provided in Section 7.

## 2. Literature review

In this section, the literature review for robust DEA as well as robust portfolio selection and optimization will be introduced. Moreover, the literature gaps and characteristics of this study will be highlighted.

### 2.1. Robust data envelopment analysis

Sadjadi & Omrani [16] were the pioneer researchers that worked on robust data envelopment analysis (RDEA) model with consideration of uncertainty on output parameters for measuring the performance of Iranian electricity distribution companies. In the last decade, the application of RDEA approach is increased more and more in different real-world problems and case studies. A more detailed classification of the most important RDEA studies is illustrated in Table 1 by considering three characteristics: DEA model, uncertainty set, and application. The characteristics of our work have also been presented in the last row of Table 1.

**Table 1:** A review of robust data envelopment analysis.

Year	Research	DEA Model	Uncertainty Set	Application
2008	Sadjadi & Omrani [16]	CCR-Input Oriented	Box & Ellipsoidal	Electricity Distribution Companies
			Box & Polyhedral	
2010	Roghanian & Foroughi [17]	CCR-Input Oriented	Box & Polyhedral	Airports
2010	Sadjadi & Omrani [18]	Bootstrap DEA	Box & Polyhedral	Telecommunication Companies
2010	Shokouhi et al. [19]	Interval DEA	BSA	
2011	Gharakhani et al. [20]	CCR-Input Oriented	Box & Ellipsoidal	High Schools
			Box & Polyhedral	
2011	Sadjadi et al. [21]	Supper Efficiency DEA	Box & Ellipsoidal	Gas Companies
2011	Sadjadi et al. [22]	Interactive DEA	Box & Polyhedral	Electricity Distribution Companies
2012	Foroughi & Esfahani [23]	CCR-Input Oriented	Box & Polyhedral	Airports
2012	Jalali Naini & Nouralizadeh [24]	CCR-Input Oriented	Box & Polyhedral	Insurance Companies
2012	Khaki et al. [25]	CCR-Input Oriented	Box & Polyhedral	Public Health Centers
2013	Omrani [26]	Common Set of Weights DEA	Box & Polyhedral	Gas Companies
2014	Shokouhi et al. [27]	Interval DEA	Box & Polyhedral	
2015	Khamseh & Zahmatkesh [28]	CCR-Input Oriented	Box & Polyhedral	Oil Facility Supporting Industry
2015	Lu [29]	BCC-Output Oriented	Ellipsoidal	Meta-Heuristics Algorithms
			Box & Polyhedral	
2015	Mardani & Salarpour [30]	Interval DEA	Box & Polyhedral	Potato Production
2016	Aghayi & Maleki [31]	Directional Distance Function	Box & Polyhedral	Power Plants / Bank Branches
2016	Aghayi et al. [32]	Common Set of Weights DEA	Box & Polyhedral	Bank Branches
2016	Ardekani et al. [33]	Network DEA	Box & Polyhedral	Electricity Power Networks
2017	Arabmaldar et al. [34]	CCR-Input Oriented	Box & Polyhedral	Forest Districts / Gas Companies
		Supper Efficiency DEA		
2017	Bayati & Sadjadi [35]	Network DEA	Box & Ellipsoidal	Electricity Power Networks
			Box & Polyhedral	
2017	Omrani & Bozorgi-Amiri [36]	CCR-Input Oriented	Box & Polyhedral	Gas Companies
2017	Shabanpour et al. [37]	CCR-Input Oriented	Box & Polyhedral	Sustainable Suppliers
2018	Ehrgott et al. [38]	BCC-Input Oriented	Ellipsoidal	Radiotherapy Design
2018	Wu & Wu [39]	CCR-Input Oriented	Ellipsoidal	Hospitals
2018	Yousefi et al. [40]	CCR-Input Oriented	Box & Polyhedral	Automotive Parts
2019	Aghayi et al. [41]	Malmquist Productivity Index	Box & Polyhedral	Bank Branches
2019	Alizadeh & Omrani [42]	CCR-Input Oriented	Box & Polyhedral	CO_2_ Laser Cutting Machine
2019	Lee & Prabhu [43]	Malmquist Productivity Index	Box & Polyhedral	Community Youth Prevention Programs
2019	Lu et al. [44]	Multi-Objective DEA	Box & Ellipsoidal	New-Energy Vehicle Manufactures
2019	Salahi et al. [45]	Russell Measure	Ellipsoidal	Banks / Flexible Manufacturing Systems
		Enhanced Russell Measure		
2019	Toloo & Mensah [46]	BCC-Input Oriented	Box & Polyhedral	Banks
2019	Yousefi et al. [47]	CCR-Input Oriented	Box & Polyhedral	Power Plants
2020	Mardani & Taki [48]	CCR-Input Oriented	Box & Polyhedral	Energy / Agriculture
2020	Mensah [49]	CCR-Input Oriented	Ellipsoidal	Banks
		Additive-Constant Returns to Scale	Box & Ellipsoidal	
2020	Salahi et al. [50]	CCR-Input Oriented	Box & Polyhedral	Gas Companies / Forest Districts
		Common Set of Weights DEA		
	**Our Work (First Phase)**	CCR-Input Oriented	Box & Polyhedral	Stock Exchange / Portfolio Selection
		CCR-Output Oriented		
		BCC-Input Oriented		
		BCC-Output Oriented		
		Additive-Constant Returns to Scale		
		Additive-Variable Returns to Scale		

### 2.2. Robust portfolio selection and optimization

There are some practical models and studies in robust portfolio selection and optimization (RPSO) problem. Ben-Tal et al. [51] initially introduced a robust model for multi-stage portfolio (asset allocation) problems. According to the applicability and effectiveness of robust optimization in investment problem, proposing and applying RPSO models have increased in recent years by many researchers [52–54]. A more detailed classification of the most important studies of robust portfolio selection and optimization is introduced in Table 2 by considering three characteristics: investment model, uncertainty set, and research feature. Also, the characteristics of our work have been illustrated in the last row of Table 2.

**Table 2:** A review of robust portfolio selection and optimization.

Year	Research	Investment Model	Uncertainty Set	Research Feature
2000	Ben-Tal et al. [51]	Asset Allocation	Ellipsoidal	Multi-Stage
2003	El Ghaoui et al. [55]	Value at Risk	Ellipsoidal	Worst Case
2003	Goldfarb and Iyengar [56]	Mean—Variance	Ellipsoidal	Simulated Data
2003	Halldórsson & Tütüncü [57]	Mean—Variance	Box	Saddle-Point Problem / Interior-Point Algorithm
2004	Tütüncü & Koenig [58]	Mean—Variance	Box	Saddle-Point Problem / Interior-Point Algorithm
2008	Bertsimas & Pachamanova [59]	Mean	Polyhedral	Multi-Period / Transaction Costs
2008	Quaranta & Zaffaroni [60]	Conditional Value at Risk	Box	Italian Market
2009	Chen and Tan [61]	Mean—Variance	Asymmetric	Interval Random Chance-Constrained Programming
2009	Zhu & Fukushima [62]	Conditional Value-at-Risk	Box	Worst-Case
			Ellipsoidal	
2011	Fonseca et al. [63]	Mean—Variance	Ellipsoidal	Currency Portfolio
2011	Gregory et al. [64]	Mean	Box & Polyhedral	Correlated / Uncorrelated / Cost of Robustness
2011	Guastaroba et al. [65]	Mean—Conditional Value-at-Risk	Box & Ellipsoidal	London Stock Exchange Market
			Box & Polyhedral	
2011	Gülpınar et al. [66]	Mean—Variance	Ellipsoidal	Discrete Asset Choice
2011	Moon & Yao [67]	Mean—Absolute Deviation	Box & Polyhedral	Three Different Time Horizons
2012	Chen & Kwon [68]	Index Tracking	Box & Polyhedral	Passive Fund Management
2012	Fonseca et al. [69]	Mean—Variance	Ellipsoidal	International Portfolio / Quanto Option
2012	Ling & Xu [70]	Mean—Variance—Option	Ellipsoidal	Option Portfolio
2012	Sadjadi et al. [71]	Mean	Box & Ellipsoidal	Cardinality Constraint / Genetic Algorithm
			Box & Polyhedral	
			Norm-Based	
2013	Ghahtarani & Najafi [72]	Mean—Systematic Risk—Beta	Box & Polyhedral	Multi-Objective / Goal Programming / Trading Constraints
2013	Gülpınar and Pachamanova [73]	Asset–Liability Management	Ellipsoidal	Pension Fund
2013	Hasuike & Katagiri [74]	Mean	Ellipsoidal	Interactive Fuzzy Satisficing Method
2014	Bandi and Bertsimas [75]	Option Pricing	Box & Polyhedral	Option Portfolio / American Option / Volatility Smile
			Norm-Based	
2014	Dai & Wen [76]	Conditional Value-at-Risk	Norm-Based	Genal Affine Data Perturbation
2015	Liu et al. [77]	Mean	Box & Polyhedral	Multi-Period / Prospect Theory / Particle Swarm Optimization
2015	Rezaie et al. [78]	Mean—Conditional Value-at-Risk	Box & Polyhedral	Ideal and Anti-Ideal Compromise Programming
2016	Gülpınar et al. [79]	Asset–Liability Management	Ellipsoidal	Investment Products with Guarantees
			Asymmetric	
2016	Li et al. [80]	Mean—Absolute Deviation	Asymmetric	Forward and Backward Deviations
2016	Wang & Cheng [81]	Mean—Variance	Box & Polyhedral	Linear Optimization Problem
			Norm-Based	
2017	Lotfi et al. [82]	Conditional Value-at-Risk	Box & Polyhedral	Buy-and-Hold Strategy
2017	Sharma et al. [83]	Omega—Conditional Value-at-Risk	Mixed	Worst Case
			Box	
			Ellipsoidal	
2018	Ghahtarani & Najafi [84]	Mean—Absolute Deviation	Box & Polyhedral	Stochastic Dominance
2018	Goli et al. [85]	Mean—Variance	Box & Polyhedral	Product Portfolio / Invasive Weed Optimization Algorithm
2019	Chen & Wei [86]	Mean—Variance	Ellipsoidal	Multi-Objective / Multi-Objective Particle Swarm Optimization
2019	Kara et al. [87]	Conditional Value-at-Risk	Parallelepiped	Robustness and Sensitivity Analysis
2019	Sehgal & Mehra [88]	Omega Ratio	Box & Polyhedral	Cutting plane algorithm
		Semi-Mean Absolute Deviation Ratio		
		Weighted Stable Tail Adjusted Return Ratio		
2020	Moghadam et al. [89]	Mean—Interval Semi-Absolute Deviation	Box & Polyhedral	Multi-Period / Prospect Theory / Genetic Algorithm / Grey Wolf Optimizer Algorithm / Butterfly Optimization Algorithm
2020	Vaezi et al. [90]	Mean	Box & Polyhedral	Genetic Algorithm / Knapsack Problem / Trading Constraints
	**Our Work (Second Phase)**	Mean—Semi Variance—Liquidity	Box & Polyhedral	Two-Phase Approach / Robust Data Envelopment Analysis
		Mean—Absolute Deviation—Liquidity		

As it can be seen in the last row of Tables 1 and 2, in this paper, a new RPSO approach will be proposed. Notably, this approach consists of two phases: the first phase is the application of robust data envelopment analysis models to qualify efficient stocks and the second phase is the application of robust portfolio optimization models in order to construct an optimal portfolio.

## 3. Nomenclatures and background

### 3.1. The nomenclatures of paper

The indices, parameters, and decision variables are described as follows:

$$\begin{array}{lll} Indices & \begin{array}{l} j\\ i\\ r\\ t \end{array} & \begin{array}{l} set\;of\;stocks\;j=1,\ldots ,n\\ the\;set\;of\;inputs\;i=1,\ldots ,m\\ set\;of\;outputs\;r=1,\ldots ,s\\ set\;of\;periods\;t=1,\ldots ,T \end{array} \end{array}$$

$$\begin{array}{lll} Parameters & \begin{array}{l} R_{tj}\\ {\bar{R}}_{j}\\ R_{E}\\ \sigma_{j}^{2}\\ \sigma_{jh}\\ {\bar{L}}_{j}\\ L_{E}\\ k\\ A_{j}\\ B_{j}\\ x_{i0}\\ y_{r0}\\ x_{ij}\\ y_{rj}\\ \Gamma \\ \Delta \\ \delta_{i} \end{array} & \begin{array}{l} return\;of\;j^{th}\;stock\;in\;i^{th}\;period\\ average\;return\;of\;j^{th}\;stock\\ benchmark\;or\;target\;level\;of\;the\;expected\;portfolio\;return\\ variance\;of\;j^{th}\;stock\\ covariance\;between\;j^{th}\;stock\;and\;h^{th}\;stock\\ average\;liquidity\;of\;j^{th}\;stock\\ benchmark\;or\;target\;level\;of\;the\;expected\;portfolio\;liquidity\\ number\;of\;authorized\;stocks\;in\;portfolio\\ minimum\;amount\;of\;the\;total\;fund\;which\;can\;be\;invested\;in\;the\;j^{th}\;stock\\ maximum\;amount\;of\;the\;total\;fund\;which\;can\;be\;invested\;in\;the\;j^{th}\;stock\\ i^{th}\;input\;of\;stock_{0}\;(the\;stock\;under\;investigation)\\ r^{th}\;output\;of\;stock_{0}\;(\;the\;stock\;under\;investigation)\\ i^{th}\;\;input\;of\;j^{th}\;stock\\ r^{th}\;\;input\;of\;j^{th}\;stock\\ level\;of\;conservatism\;\left(budget\;of\;uncertainty\right)\\ perturbation\;of\;uncertain\;parameters\\ confidence\;level\;for\;satisfying\;the\;i^{th}\;constraint \end{array} \end{array}$$

$$\begin{array}{lll} Decision\;Variables & \begin{array}{l} \omega_{j}\\ \tau_{j}\\ \xi_{t}\\ \zeta_{t}\\ u_{r}\\ v_{i}\\ w_{0} \end{array} & \begin{array}{l} weight\;of\;j^{th}\;stock\;in\;portfolio\\ binary\;variable\;which\;will\;be\;one\;if\;any\;of\;j^{th}\;stock\;is\;held\;and\;zero\;otherwise\\ semi\;variance\;of\;portfolio\;in\;t^{th}\;period\\ absolute\;deviation\;of\;portfolio\;t^{th}\;inperiod\\ weight\;for\;the\;r^{th}\;output\\ weight\;for\;the\;i^{th}\;input\\ returns\;to\;scale\;of\;stock_{0}\;is\;the\;stock\;under\;investigation \end{array} \end{array}$$

### 3.2. Classic portfolio models and risk measures

The first method in portfolio selection is proposed by Markowitz [4]. The mean-variance (MV) model for solving the portfolio selection problem is as Model (1):

$$\begin{array}{ll} & \Theta^{\text{MV}}\\ \text{Min} & \sum_{j=1}^{n}\omega_{j}^{2}\sigma_{j}^{2}+2\sum_{j=1}^{n-1}\sum_{h=j+1}^{n}\omega_{j}\omega_{h}\sigma_{jh}=\;\sum_{j=1}^{n}\sum_{h=1}^{n}\omega_{j}\omega_{h}\sigma_{jh}\\ \text{S}.\text{t}. & \sum_{j=1}^{n}{\bar{R}}_{j}\omega_{j}\geq R_{E}\\ & \sum_{j=1}^{n}\omega_{j}=1\\ & \omega_{j}\geq 0,\;\forall j \end{array}$$ (1)

As shown in Model (1), the variance criterion is used as a risk measure for portfolio. It should be explained that variance as a risk measure for portfolio selection penalizes both returns above and below expected return. Markowitz [91] suggested semi variance (SV) as a downside risk measure that quantifies possibilities of return below the expected return. The definition of semi variance risk measure is as Eq (2):

$$SV=E({\left(Max\{0,R_{E}-\sum_{j=1}^{n}R_{ij}\omega_{j}\}\right)}^{2})=\left\{ \begin{array}{ll} {\left(R_{E}-\sum_{j=1}^{n}R_{ij}\omega_{j}\right)}^{2} & if\;R_{E}-\sum_{j=1}^{n}R_{ij}\omega_{j}>0\;;\\ 0 & if\;R_{E}-\sum_{j=1}^{n}R_{ij}\omega_{j}\leq 0. \end{array}\right.$$ (2)

To solve the mean-variance model, DMs need the covariance matrix that estimation of this matrix is difficult with the real-world data, but by using of the mean- semi variance (MSV) model, it is not required to compute the covariance matrix and the joint distribution of stocks is needed to be computed.

Since the original Markowitz’s [4] model is a quadratic programming (QP) model and it is difficult to be solved for large data sets, Konno & Yamazaki [92] proposed absolute deviation (AD) instead of variance as a risk measure for portfolio selection. The mean- absolute deviation model (MAD) is a linear programming (LP) model and reduce computational time. The definition of absolute deviation is as Eq (3):

$$AD=|R_{E}-\sum_{j=1}^{n}R_{ij}\omega_{j}|=\left\{ \begin{array}{l} R_{E}-\sum_{j=1}^{n}R_{ij}\omega_{j}\;if\;R_{E}-\sum_{j=1}^{n}R_{ij}\omega_{j}>0\;;\\ \sum_{j=1}^{n}R_{ij}\omega_{j}-R_{E}\;if\;R_{E}-\sum_{j=1}^{n}R_{ij}\omega_{j}\leq 0. \end{array}\right.$$ (3)

This risk measure quantifies the deviation from the expected return and by using MAD model, it is not required to compute the covariance matrix.

### 3.3. Data envelopment analysis

Data envelopment analysis was proposed by Charnes et al. [93] for the first time and it is based on Farrell’s [94] idea. This methodology is a non-parametric technique for performance evaluation and ranking the homogeneous decision-making units. Charnes et al. [93] proposed the first DEA model that based on the constant returns to scale (CRS) assumption and called the CCR model. Then, Banker et al. [95] developed CCR model based on the variable returns to scale (VRS) assumption and called the BCC model. The CCR and BCC models are radial projection constructs. Charnes et al. [96] proposed the DEA model by considering simultaneously both input minimization and output maximization which is called Additive (ADD) model. It is worth noting that CCR, BCC and Additive models are radial, radial and non-radial models, respectively.

$$\begin{array}{ll} & \Theta_{\text{Classic}}^{\text{CCR}-\text{IO}}\\ \text{Max} & \sum_{r=1}^{s}y_{r0}u_{r}\\ \text{S}.\text{t}. & \sum_{i=1}^{m}x_{i0}v_{i}=1\\ & \sum_{r=1}^{s}y_{rj}u_{r}-\sum_{i=1}^{m}x_{ij}v_{i}\leq 0,\;\forall j\\ & u_{r},v_{i}\geq 0,\;\forall r,i \end{array}$$ (4)

$$\begin{array}{ll} & \Theta_{\text{Classic}}^{\text{CCR}-\text{OO}}\\ \text{Min} & \sum_{i=1}^{m}x_{i0}v_{i}\\ \text{S}.\text{t}. & \sum_{r=1}^{s}y_{r0}u_{r}=1\\ & \sum_{r=1}^{s}y_{rj}u_{r}-\sum_{i=1}^{m}x_{ij}v_{i}\leq 0,\;\forall j\\ & u_{r},v_{i}\geq 0,\;\forall r,i \end{array}$$ (5)

$$\begin{array}{ll} & \Theta_{\text{Classic}}^{\text{BCC}-\text{IO}}\\ \text{Max} & \sum_{r=1}^{s}y_{r0}u_{r}+w_{0}\\ \text{S}.\text{t}. & \sum_{i=1}^{m}x_{i0}v_{i}=1\\ & \sum_{r=1}^{s}y_{rj}u_{r}-\sum_{i=1}^{m}x_{ij}v_{i}+w_{0}\leq 0,\;\forall j\\ & u_{r},v_{i}\geq 0,\;\forall r,i \end{array}$$ (6)

$$\begin{array}{ll} & \Theta_{\text{Classic}}^{\text{BCC}-\text{OO}}\\ \text{Min} & \sum_{i=1}^{m}x_{i0}v_{i}-w_{0}\\ \text{S}.\text{t}. & \sum_{r=1}^{s}y_{r0}u_{r}=1\\ & \sum_{r=1}^{s}y_{rj}u_{r}-\sum_{i=1}^{m}x_{ij}v_{i}+w_{0}\leq 0,\;\forall j\\ & u_{r},v_{i}\geq 0,\;\forall r,i \end{array}$$ (7)

$$\begin{array}{ll} & \Theta_{\text{Classic}}^{\text{ADD-CRS}}\\ \text{Min} & \sum_{i=1}^{m}x_{i0}v_{i}-\sum_{r=1}^{s}y_{r0}u_{r}\\ \text{S}.\text{t}. & -\sum_{r=1}^{s}y_{rj}u_{r}+\sum_{i=1}^{m}x_{ij}v_{i}\geq 0,\;\forall j\\ & u_{r}\geq 1,\;\forall r\\ & v_{i}\geq 1,\;\forall i \end{array}$$ (8)

$$\begin{array}{ll} & \Theta_{\text{Classic}}^{\text{ADD-VRS}}\\ \text{Min} & \sum_{i=1}^{m}x_{i0}v_{i}-\sum_{r=1}^{s}y_{r0}u_{r}-w_{0}\\ \text{S}.\text{t}. & -\sum_{r=1}^{s}y_{rj}u_{r}+\sum_{i=1}^{m}x_{ij}v_{i}-w_{0}\geq 0,\;\forall j\\ & u_{r}\geq 1,\;\forall r\\ & v_{i}\geq 1,\;\forall i \end{array}$$ (9)

With respect to CCR, BCC and Additive models are basic and popular DEA models, in this research, input-oriented CCR (CCR-IO) model, output-oriented CCR (CCR-OO) model, input-oriented BCC (BCC-IO) model, output-oriented BCC (BCC-OO) model, Additive model with constant returns to scale (ADD-CRS) and Additive model with variable returns to scale (ADD-VRS) will be applied. The multiplier form of CCR-IO, CCR-OO, BCC-IO, BCC-OO, ADD-CRS and ADD-VRS models are introduced in Models (4) to (9), respectively.

### 3.4. Robust optimization

In real cases, generally, the inputs and outputs of DEA models are tainted by uncertainty [97–105]. The imprecision of the input parameters increases when there is a low access to reliable historical data. In this condition, it is important to protect the robustness of the solution obtained from the DEA model; otherwise, the efficiency and ranking of the concerned DMUs may become unreliable and consequently significant costs may impose on different stakeholders. To prevent such undesirable outcome robust optimization methods can be employed [106]. Notably, a solution to a DEA model is said to be robust if it remains feasible for almost all possible values of uncertain parameters and the corresponding ranking should have minimum variation for all possible values of imprecise parameters. Here, a hard-worst-case robust optimization approach is applied to cope with uncertain parameters in the DEA model [107]. This approach does not need significant historical data and therefore it can be applied in almost all of the real-life DEA problems. In addition, this method assures the feasibility of the DEA model solution for all possible values of uncertain parameters in the assumed convex uncertainty set. Soyster [108], Ben-Tal & Nemirovski [109] and Bertsimas & Sim [110] presented a popular and main robust optimization approach in convex uncertainty set.

In robust optimization method, for dealing with uncertainty in data, consider a particular constraint *a* of a nominal model and let Λ_*a*_ represent the set of coefficients in constraint *a* that are subject to uncertainty. It should be noted that each entry *α*_*ab*_,*b* ∈ Λ_*a*_ is modeled as a symmetric and bounded random variable which takes values in $\left[\alpha_{ab}-{\hat{\alpha }}_{ab},\alpha_{ab}+{\hat{\alpha }}_{ab}\right)$. The central of this interval at the point *α*_*ab*_ is a nominal value and ${\hat{\alpha }}_{ab}$ is the perturbation of uncertain parameters *α*_*ab*_,*b* ∈ Λ_*a*_. Finally, robust counterpart of constraint *a* based on Soyster [108], Ben-Tal & Nemirovski [109] and Bertsimas & Sim [110] robust optimization approaches are proposed as Eqs (10) to (12), respectively:

$$\begin{array}{l} Soyster\\ \left\{ \begin{array}{l} \sum_{b}\alpha_{ab}\;\varphi_{b}+\sum_{b\in \Lambda_{a}}{\hat{\alpha }}_{ab}\phi_{b}\leq \beta_{a},\;\forall a\\ -\phi_{b}\leq \varphi_{b}\leq \phi_{b},\;\forall b\\ \phi \geq 0 \end{array}\right. \end{array}$$ (10)

$$\begin{array}{ll} \begin{array}{l} Robust\;Counterpart\;of\\ \sum_{b}{\tilde{\alpha }}_{ab}\varphi_{b}\leq \beta_{a},\;\forall a \end{array} & \;\begin{array}{l} Ben-Tal\;\&\;Nemirovski\\ \left\{ \begin{array}{l} \sum_{b}\alpha_{ab}\;\varphi_{b}+\sum_{b\in \Lambda_{a}}{\hat{\alpha }}_{ab}\phi_{ab}+\Omega_{a}\;\sqrt{\sum_{b\in \Lambda_{a}}{\hat{\alpha }}_{ab}^{2}\;\sigma_{ab}^{2}}\leq \beta_{a},\;\forall a\\ -\phi_{ab}\leq \varphi_{b}-\sigma_{ab}\leq \phi_{ab},\;\forall a,b\in \Lambda_{a}\\ \phi \geq 0 \end{array}\right. \end{array} \end{array}$$ (11)

$$\begin{array}{l} Bertsimas\;\&\;Sim\\ \left\{ \begin{array}{l} \sum_{b}\alpha_{ab}\;\varphi_{b}+Z_{a}\Gamma_{a}+\sum_{b\in \Lambda_{a}}P_{ab}\leq \beta_{a},\;\forall a\\ Z_{a}+P_{ab}\geq {\hat{\alpha }}_{ab}\phi_{b},\;\forall a,b\in \Lambda_{a}\\ -\phi_{b}\leq \varphi_{b}\leq \phi_{b},\;\forall a,b\in \Lambda_{a}\\ Z,P,\phi \geq 0 \end{array}\right. \end{array}$$ (12)

It is worth mentioning that robust optimization approach of Soyster [108] is too conservative. Ben-Tal & Nemirovski [109] proposed a robust approach but their robust counterpart is nonlinear programming (NLP) which can be problematic in the real-world problems although the model can adjust the conservatism by parameter Ω. Bertsimas and Sim’s [110] robust approach can flexibly adjust the level of conservatism of the robust solutions by parameter Γ and robust counterpart in their approach is linear programming (LP) [111–114]. With respect to this feature and linearity of robust counterpart in Bertsimas and Sim’s [110] robust approach, this approach will be used in this paper for dealing with uncertainty in all models. Please note that RO approaches of Soyster [108], Ben-Tal & Nemirovski [109] and Bertsimas & Sim [110] are presented based on “box”, “box & ellipsoidal”, and “box & polyhedral” uncertainty sets, respectively.

## 4. The proposed robust approach for portfolio selection and optimization problem

**Fig 1:**
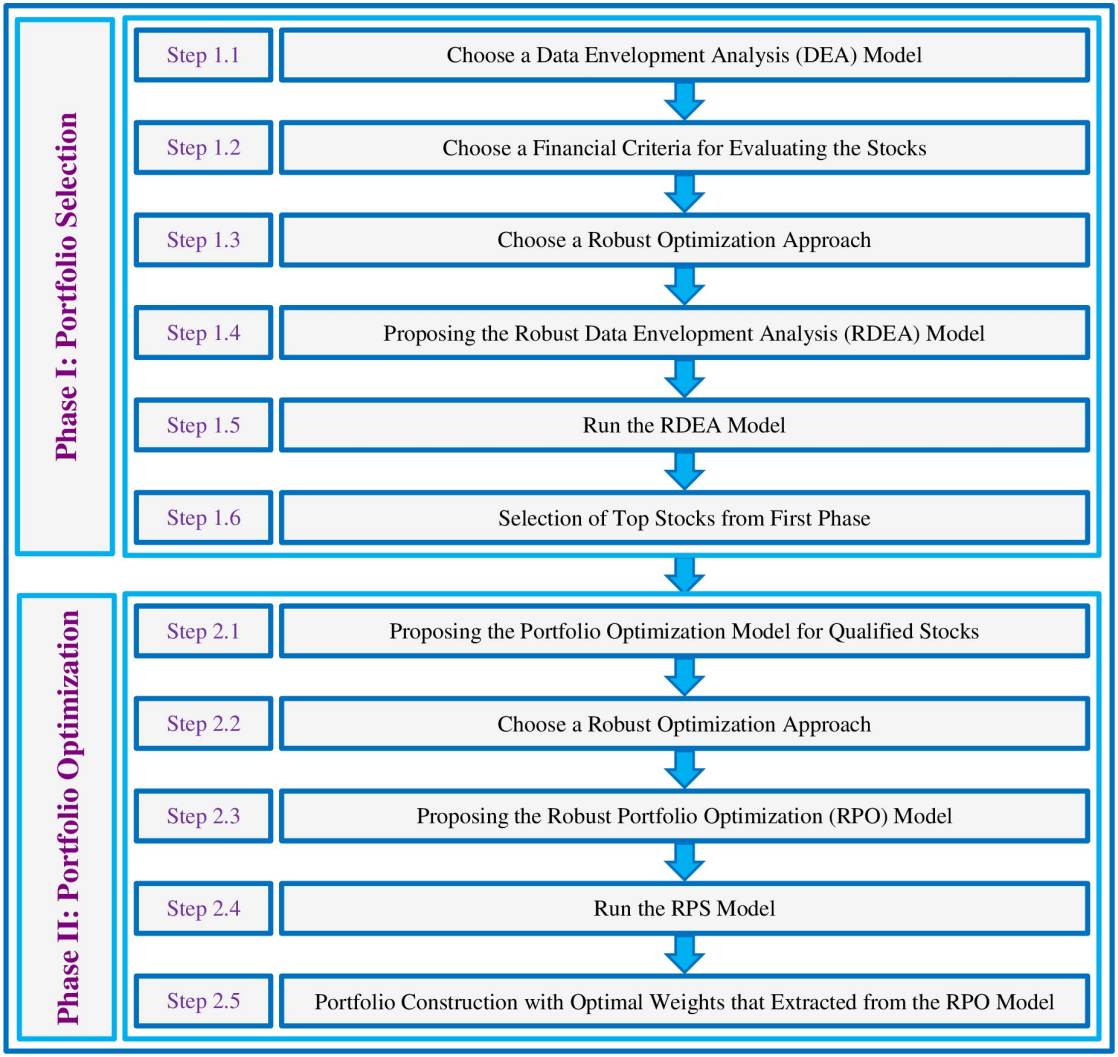
The methodology of proposed two-phase robust portfolio selection and optimization approach.

In this section, the robust approach for portfolio construction problem in the financial markets is presented. This approach contains two phases that in continuous, steps of each phase, thoroughly are explained. Fig 1 presents a schematic summary of all steps in two-phase robust portfolio construction approach of this paper.

### 4.1. Phase I: Portfolio selection

In this phase during 6 steps, the performance of all stocks that investors can invest in them, are evaluated and measured. At the end of this phase, only the stocks that pass the filter of the investor are qualified to be a candidate that can be invested in the second phase.

#### Step 1.1. Choose a Data Envelopment Analysis (DEA) model

In the first step of phase 1, the data envelopment analysis models are chosen to evaluate the stocks. In this paper, CCR-IO, CCR-OO, BCC-IO, BCC-OO, ADD-CRS and ADD-VRS models are selected. Notably, all of DEA models that are used in this study, are presented in the Subsection 3.3.

#### Step 1.2. Choose a financial criteria for evaluating the stocks

In the second step of phase 1, financial criteria for evaluation of stocks are chosen from different perspectives that contains of return, risk, profitability, liquidity, leverage, valuation and growth. Based on literature review, expert opinion and Delphi method, inputs and outputs of DEA models are as shown in Table 3.

**Table 3:** The inputs and outputs of DEA models.

	Financial Criteria	Symbol	Description
Inputs	Price to Earnings Ratio (P/E)	I (1)	Stock price divided by net income per share
	Quick Ratio	I (2)	Total current assets minus inventory divided by total current liabilities
	Solvency Ratio-II	I (3)	Total liability divided by shareholders equity
	Beta (*β*)	I (4)	Systematic Risk
	Standard Deviation (*σ*)	I (5)	Non-Systematic Risk
Outputs	Earnings per Share (EPS)	O (1)	Net income minus dividends divided by common shares
	Rate of Return	O (2)	Proportion of gain or loss on an investment over a specified period
	Rate of Liquidity	O (3)	Degree which presents stock ability to be bought or sold in the market quickly
	Earnings per Share Growth Rate	O (4)	Current quarters EPS divided by the previous quarters EPS minus one

#### Step 1.3. Choose a robust optimization approach

In the third step of phase 1, with respect to weaknesses and strengths of Soyster [108], Ben-Tal & Nemirovski [109] and Bertsimas & Sim [110] robust approaches, the Bertsimas & Sim’s [110] (B&S) robust approach are selected for dealing with uncertain parameters in DEA models. It should be noted that the formulation of robust counterpart in the B&S robust approach are presented in Subsection 3.4.

#### Step 1.4. Proposing the Robust Data Envelopment Analysis (RDEA) model

In the fourth step of phase 1, robust data envelopment analysis models are proposed. This step is the most important step in the first phase. In order to consider the uncertainty of input and output parameters in DEA models based on Bertsimas & Sim’s [110] robust approach, primarily all of the constraints, to become less than or equal constraints. In each of the CCR-IO, CCR-OO, BCC-IO and BCC-OO models, how to convert the equal constraint to less than or equal constraints, will be discussed in the following, respectively.

The compact form (CF) of CCR-IO model is as Model (13). If *vx*_0_ = 1 become to *vx*_0_ ≤ 1, the optimal solution does not change.

$$\Theta_{\text{Classic}(\text{CF})}^{\;\text{CCR}-\text{IO}}$$

$$\begin{array}{ll} \text{Max} & uy_{0}\\ \text{S}.\text{t}. & vx_{0}=1\\ & uy_{j}-vx_{j}\leq 0,\;\forall j\\ & u,v\geq 0 \end{array}$$ (13)

$$\begin{array}{ll} \text{Max} & uy_{0}\\ \text{S}.\text{t}. & vx_{0}\leq 1\\ & uy_{j}-vx_{j}\leq 0,\;\forall j\\ & u,v\geq 0 \end{array}$$ (14)

**Proposition 1**. The optimal solution of Model (13) is equal to Model (14).

**Proof**. Assume that the optimal solution of Model (14) is $\left(\bar{u},\bar{v}\right)$. By contradiction, suppose that $\bar{v}x_{0}<1$ (it should be noted that $\bar{v}x_{0}>0$). $\left(\hat{u},\hat{v}\right)$ are considered as $\hat{u}=\bar{u}/\bar{v}x_{0}$ and $\hat{v}=\bar{v}/\bar{v}x_{0}$. Because of $\hat{u}y_{j}-\hat{v}x_{j}=\left(\bar{u}y_{j}-\bar{v}x_{j}\right)/\bar{v}x_{0}\leq 0$ (with respect to $1/\bar{v}x_{0}>0$ and $\bar{u}y_{j}-\bar{v}x_{j}\leq 0$), $\hat{v}x_{0}=\left(\bar{v}x_{0}\right)/\bar{v}x_{0}=1$, $\hat{u}\geq 0$ and $\hat{v}\geq 0$, $\left(\hat{u},\hat{v}\right)$ are the feasible solution of Model (14). Also, in the objective function $\hat{u}y_{0}=\left(\bar{u}y_{0}\right)/\bar{v}x_{0}$, with respect to suppose that $\bar{v}x_{0}<1$, thus $1/\bar{v}x_{0}>1$ and finally $\hat{u}y_{0}>\bar{u}y_{0}$ that this is contradicts with optimality of $\left(\bar{u},\bar{v}\right)$. So, at any optimal solution of Model (14), always $\bar{v}x_{0}=1$.

The compact form of CCR-OO model is as Model (15). If *uy*_0_ = 1 become to *uy*_0_ ≥ 1, the optimal solution does not change.

$$\Theta_{\text{Classic}(\text{CF})}^{\;\text{CCR}-\text{OO}}$$

$$\begin{array}{ll} \text{Min} & vx_{0}\\ \text{S}.\text{t}. & uy_{0}=1\\ & uy_{j}-vx_{j}\leq 0,\;\forall j\\ & u,v\geq 0 \end{array}$$ (15)

$$\begin{array}{ll} \text{Min} & vx_{0}\\ \text{S}.\text{t}. & uy_{0}\geq 1\\ & uy_{j}-vx_{j}\leq 0,\;\forall j\\ & u,v\geq 0 \end{array}$$ (16)

**Proposition 2**. The optimal solution of Model (15) is equal to Model (16).

**Proof**. Assume that the optimal solution of Model (16) is $\left(\bar{u},\bar{v}\right)$. By contradiction, suppose that $\bar{u}y_{0}>1$. $\left(\hat{u},\hat{v}\right)$ are considered as $\hat{u}=\bar{u}/\bar{u}y_{0}$ and $\hat{v}=\bar{v}/\bar{u}y_{0}$. Because of $\hat{u}y_{j}-\hat{v}x_{j}=\left(\bar{u}y_{j}-\bar{v}x_{j}\right)/\bar{u}y_{0}\leq 0$ (with respect to $1/\bar{u}y_{0}>0$ and $\bar{u}y_{j}-\bar{v}x_{j}\leq 0$), $\hat{u}y_{0}=\left(\bar{u}y_{0}\right)/\bar{u}y_{0}=1$, $\hat{u}\geq 0$ and $\hat{v}\geq 0$, $\left(\hat{u},\hat{v}\right)$ is the feasible solution of Model (16). Also, in the objective function $\hat{v}x_{0}=\left(\bar{v}x_{0}\right)/\bar{u}y_{0}$, with respect to suppose that $\bar{u}y_{0}>1$, thus $1/\bar{u}y_{0}<1$ and finally $\hat{v}x_{0}<\bar{v}x_{0}$ that this is contradicts with optimality of $\left(\bar{u},\bar{v}\right)$. So, at any optimal solution of Model (16), always $\bar{u}y_{0}=1$.

The compact form of BCC-IO Model is as Model (17). If *vx*_0_ = 1 become to *vx*_0_ ≤ 1, the optimal solution does not change.

$$\Theta_{\text{Classic}(\text{CF})}^{\;\text{BCC}-\text{IO}}$$

$$\begin{array}{ll} \text{Max} & uy_{0}+w_{0}\\ \text{S}.\text{t}. & vx_{0}=1\\ & uy_{j}-vx_{j}+w_{0}\leq 0,\;\forall j\\ & u,v\geq 0 \end{array}$$ (17)

$$\begin{array}{ll} \text{Max} & uy_{0}+w_{0}\\ \text{S}.\text{t}. & vx_{0}\leq 1\\ & uy_{j}-vx_{j}+w_{0}\leq 0,\;\forall j\\ & u,v\geq 0 \end{array}$$ (18)

**Proposition 3**. The optimal solution of Model (17) is equal to Model (18).

**Proof**. Assume that the optimal solution of Model (18) is $\left(\bar{u},\bar{v},{\bar{w}}_{0}\right)$. By contradiction, suppose that $\bar{v}x_{0}<1$ (it should be noted that $\bar{v}x_{0}>0$). $\left(\hat{u},\hat{v},{\hat{w}}_{0}\right)$ are considered as $\hat{u}=\bar{u}/\bar{v}x_{0}$, $\hat{v}=\bar{v}/\bar{v}x_{0}$ and ${\hat{w}}_{0}={\bar{w}}_{0}/\bar{v}x_{0}$. Because of $\hat{u}y_{j}-\hat{v}x_{j}+{\hat{w}}_{0}=\left(\bar{u}y_{j}-\bar{v}x_{j}+{\bar{w}}_{0}\right)/\bar{v}x_{0}\leq 0$ (with respect to $1/\bar{v}x_{0}>0$ and $\bar{u}y_{j}-\bar{v}x_{j}+{\bar{w}}_{0}\leq 0$), $\hat{v}x_{0}=\left(\bar{v}x_{0}\right)/\bar{v}x_{0}=1$, $\hat{u}\geq 0$ and $\hat{v}\geq 0$, $\left(\hat{u},\hat{v},{\hat{w}}_{0}\right)$ is the feasible solution of Model (18). Also, in the objective function $\hat{u}y_{0}+{\hat{w}}_{0}=\left(\bar{u}y_{0}+{\bar{w}}_{0}\right)/\bar{v}x_{0}$, with respect to suppose that $\bar{v}x_{0}<1$, thus $1/\bar{v}x_{0}>1$ and finally $\hat{u}y_{0}+{\hat{w}}_{0}>\bar{u}y_{0}+{\bar{w}}_{0}$ that this is contradicts with optimality of $\left(\bar{u},\bar{v},{\bar{w}}_{0}\right)$. So, at any optimal solution of Model (18), always $\bar{v}x_{0}=1$.

The compact form of BCC-OO Model is as Model (19). If *uy*_0_ = 1 become to *uy*_0_ ≥ 1, the optimal solution does not change.

$$\Theta_{\text{Classic}(\text{CF})}^{\;\text{BCC}-\text{OO}}$$

$$\begin{array}{ll} \text{Min} & vx_{0}-w_{0}\\ \text{S}.\text{t}. & uy_{0}=1\\ & uy_{j}-vx_{j}+w_{0}\leq 0,\;\forall j\\ & u,v\geq 0 \end{array}$$ (19)

$$\begin{array}{ll} \text{Min} & vx_{0}-w_{0}\\ \text{S}.\text{t}. & uy_{0}\geq 1\\ & uy_{j}-vx_{j}+w_{0}\leq 0,\;\forall j\\ & u,v\geq 0 \end{array}$$ (20)

**Proposition 4**. The optimal solution of Model (19) is equal to Model (20).

**Proof**. Assume that the optimal solution of Model (20) is $\left(\bar{u},\bar{v},{\bar{w}}_{0}\right)$. By contradiction, suppose that $\bar{u}y_{0}>1$. $\left(\hat{u},\hat{v},{\hat{w}}_{0}\right)$ are considered as $\hat{u}=\bar{u}/\bar{u}y_{0}$, $\hat{v}=\bar{v}/\bar{u}y_{0}$ and ${\hat{w}}_{0}={\bar{w}}_{0}/\bar{u}y_{0}$. Because of $\hat{u}y_{j}-\hat{v}x_{j}+{\hat{w}}_{0}=\left(\bar{u}y_{j}-\bar{v}x_{j}+{\bar{w}}_{0}\right)/\bar{u}y_{0}\leq 0$ (with respect to $1/\bar{u}y_{0}>0$ and $\bar{u}y_{j}-\bar{v}x_{j}+{\bar{w}}_{0}\leq 0$), $\hat{u}y_{0}=\left(\bar{u}y_{0}\right)/\bar{u}y_{0}=1$, $\hat{u}\geq 0$ and $\hat{v}\geq 0$, $\left(\hat{u},\hat{v},{\hat{w}}_{0}\right)$ is the feasible solution of Model (20). Also, in the objective function $\hat{v}x_{0}-{\hat{w}}_{0}=\left(\bar{v}x_{0}-{\bar{w}}_{0}\right)/\bar{u}y_{0}$, with respect to suppose that $\bar{u}y_{0}>1$, thus $1/\bar{u}y_{0}<1$ and finally $\hat{v}x_{0}-{\hat{w}}_{0}<\bar{v}x_{0}-{\bar{w}}_{0}$ that this is contradicts with optimality of $\left(\bar{u},\bar{v},{\bar{w}}_{0}\right)$. So, at any optimal solution of Model (20), always $\bar{u}y_{0}=1$.

Now, according to B&S robust approach, all the robust DEA models in this paper that contain RCCR-IO, RCCR-OO, RBCC-IO, RBCC-OO, RADD-CRS, and RADD-VRS are presented as Models (21) to (26), respectively:

$$\begin{array}{ll} & \Theta_{\text{Robust}}^{\text{CCR}-\text{IO}}\\ \text{Max} & \Psi \\ \text{S}.\text{t}. & \Psi -\sum_{r=1}^{s}y_{r0}u_{r}+Z_{0}^{y}\Gamma_{0}^{y}+\sum_{r=1}^{s}P_{r0}^{y}\leq 0\\ & \sum_{i=1}^{m}x_{i0}v_{i}+Z_{0}^{x}\Gamma_{0}^{x}+\sum_{i=1}^{m}P_{i0}^{x}\leq 1\\ & \sum_{r=1}^{s}y_{rj}u_{r}-\sum_{i=1}^{m}x_{ij}v_{i}+Z_{j}\Gamma_{j}+\sum_{r=1}^{s}P_{rj}^{y}+\sum_{i=1}^{m}P_{ij}^{x}\leq 0,\;\forall j\\ & Z_{0}^{y}+P_{r0}^{y}\geq \Delta y_{r0}u_{r},\;\forall r\\ & Z_{0}^{x}+P_{i0}^{x}\geq \Delta x_{i0}v_{i},\;\forall i\\ & Z_{j}+P_{rj}^{y}\geq \Delta y_{rj}u_{r},\;\forall j,r\\ & Z_{j}+P_{ij}^{x}\geq \Delta x_{ij}v_{i},\;\forall j,i\\ & Z_{0}^{x},Z_{0}^{y},Z_{j},P_{r0}^{y},P_{rj}^{y},P_{i0}^{x},P_{ij}^{x},u_{r},v_{i}\geq 0,\;\forall j,r,i \end{array}$$ (21)

$$\begin{array}{ll} & \Theta_{\text{Robust}}^{\text{CCR}-\text{OO}}\\ \text{Min} & \Psi \\ \text{S}.\text{t}. & \sum_{i=1}^{m}x_{i0}v_{i}+Z_{0}^{x}\Gamma_{0}^{x}+\sum_{i=1}^{m}P_{i0}^{x}-\Psi \leq 0\\ & -\sum_{r=1}^{s}y_{r0}u_{r}+Z_{0}^{y}\Gamma_{0}^{y}+\sum_{r=1}^{s}P_{r0}^{y}\leq -1\\ & \sum_{r=1}^{s}y_{rj}u_{r}-\sum_{i=1}^{m}x_{ij}v_{i}+Z_{j}\Gamma_{j}+\sum_{r=1}^{s}P_{rj}^{y}+\sum_{i=1}^{m}P_{ij}^{x}\leq 0,\;\forall j\\ & Z_{0}^{x}+P_{i0}^{x}\geq \Delta x_{i0}v_{i},\;\forall i\\ & Z_{0}^{y}+P_{r0}^{y}\geq \Delta y_{r0}u_{r},\;\forall r\\ & Z_{j}+P_{rj}^{y}\geq \Delta y_{rj}u_{r},\;\forall j,r\\ & Z_{j}+P_{ij}^{x}\geq \Delta x_{ij}v_{i},\;\forall j,i\\ & Z_{0}^{x},Z_{0}^{y},Z_{j},P_{r0}^{y},P_{rj}^{y},P_{i0}^{x},P_{ij}^{x},u_{r},v_{i}\geq 0,\;\forall j,r,i \end{array}$$ (22)

$$\begin{array}{ll} & \Theta_{\text{Robust}}^{\text{BCC}-\text{IO}}\\ \text{Min} & \Psi \\ \text{S}.\text{t}. & \Psi -\sum_{r=1}^{s}y_{r0}u_{r}-w_{0}+Z_{0}^{y}\Gamma_{0}^{y}+\sum_{r=1}^{s}P_{r0}^{y}\leq 0\\ & \sum_{i=1}^{m}x_{i0}v_{i}+Z_{0}^{x}\Gamma_{0}^{x}+\sum_{i=1}^{m}P_{i0}^{x}\leq 1\\ & \sum_{r=1}^{s}y_{rj}u_{r}-\sum_{i=1}^{m}x_{ij}v_{i}+w_{0}+Z_{j}\Gamma_{j}+\sum_{r=1}^{s}P_{rj}^{y}+\sum_{i=1}^{m}P_{ij}^{x}\leq 0,\;\forall j\\ & Z_{0}^{y}+P_{r0}^{y}\geq \Delta y_{r0}u_{r},\;\forall r\\ & Z_{0}^{x}+P_{i0}^{x}\geq \Delta x_{i0}v_{i},\;\forall i\\ & Z_{j}+P_{rj}^{y}\geq \Delta y_{rj}u_{r},\;\forall j,r\\ & Z_{j}+P_{ij}^{x}\geq \Delta x_{ij}v_{i},\;\forall j,i\\ & Z_{0}^{x},Z_{0}^{y},Z_{j},P_{r0}^{y},P_{rj}^{y},P_{i0}^{x},P_{ij}^{x},u_{r},v_{i}\geq 0,\;\forall j,r,i \end{array}$$ (23)

$$\begin{array}{ll} & \Theta_{\text{Robust}}^{\text{BCC}-\text{OO}}\\ \text{Min} & \Psi \\ \text{S}.\text{t}. & \sum_{i=1}^{m}x_{i0}v_{i}-w_{0}+Z_{0}^{x}\Gamma_{0}^{x}+\sum_{i=1}^{m}P_{i0}^{x}-\Psi \leq 0\\ & -\sum_{r=1}^{s}y_{r0}u_{r}+Z_{0}^{y}\Gamma_{0}^{y}+\sum_{r=1}^{s}P_{r0}^{y}\leq -1\\ & \sum_{r=1}^{s}y_{rj}u_{r}-\sum_{i=1}^{m}x_{ij}v_{i}+w_{0}+Z_{j}\Gamma_{j}+\sum_{r=1}^{s}P_{rj}^{y}+\sum_{i=1}^{m}P_{ij}^{x}\leq 0,\;\forall j\\ & Z_{0}^{x}+P_{i0}^{x}\geq \Delta x_{i0}v_{i},\;\forall i\\ & Z_{0}^{y}+P_{r0}^{y}\geq \Delta y_{r0}u_{r},\;\forall r\\ & Z_{j}+P_{rj}^{y}\geq \Delta y_{rj}u_{r},\;\forall j,r\\ & Z_{j}+P_{ij}^{x}\geq \Delta x_{ij}v_{i},\;\forall j,i\\ & Z_{0}^{x},Z_{0}^{y},Z_{j},P_{r0}^{y},P_{rj}^{y},P_{i0}^{x},P_{ij}^{x},u_{r},v_{i}\geq 0,\;\forall j,r,i \end{array}$$ (24)

$$\begin{array}{ll} & \Theta_{\text{Robust}}^{\text{ADD-CRS}}\\ \text{Min} & \Psi \\ \text{S}.\text{t}. & -\;\sum_{r=1}^{s}y_{r0}u_{r}+\sum_{i=1}^{m}x_{i0}v_{i}+Z_{0}\Gamma_{0}+\sum_{r=1}^{s}P_{r0}^{y}+\sum_{i=1}^{m}P_{i0}^{x}-\Psi \leq 0\\ & \sum_{r=1}^{s}y_{rj}u_{r}-\sum_{i=1}^{m}x_{ij}v_{i}+Z_{j}\Gamma_{j}+\sum_{r=1}^{s}P_{rj}^{y}+\sum_{i=1}^{m}P_{ij}^{x}\leq 0,\;\forall j\\ & u_{r}\geq 1,\;\forall r\\ & v_{i}\geq 1,\;\forall i\\ & Z_{0}+P_{r0}^{y}\geq \Delta y_{r0}u_{r},\;\forall r\\ & Z_{0}+P_{i0}^{x}\geq \Delta x_{i0}v_{i},\;\forall i\\ & Z_{j}+P_{rj}^{y}\geq \Delta y_{rj}u_{r},\;\forall j,r\\ & Z_{j}+P_{ij}^{x}\geq \Delta x_{ij}v_{i},\;\forall j,i\\ & Z_{0},Z_{j},P_{r0}^{y},P_{rj}^{y},P_{i0}^{x},P_{ij}^{x},u_{r},v_{i}\geq 0,\;\forall j,r,i \end{array}$$ (25)

$$\begin{array}{ll} & \Theta_{\text{Robust}}^{\text{ADD-VRS}}\\ \text{Min} & \Psi \\ \text{S}.\text{t}. & -\;\sum_{r=1}^{s}y_{r0}u_{r}+\sum_{i=1}^{m}x_{i0}v_{i}-w_{0}+Z_{0}\Gamma_{0}+\sum_{r=1}^{s}P_{r0}^{y}+\sum_{i=1}^{m}P_{i0}^{x}-\Psi \leq 0\\ & \sum_{r=1}^{s}y_{rj}u_{r}-\sum_{i=1}^{m}x_{ij}v_{i}+w_{0}+Z_{j}\Gamma_{j}+\sum_{r=1}^{s}P_{rj}^{y}+\sum_{i=1}^{m}P_{ij}^{x}\leq 0,\;\forall j\\ & u_{r}\geq 1,\;\forall r\\ & v_{i}\geq 1,\;\forall i\\ & Z_{0}+P_{r0}^{y}\geq \Delta y_{r0}u_{r},\;\forall r\\ & Z_{0}+P_{i0}^{x}\geq \Delta x_{i0}v_{i},\;\forall i\\ & Z_{j}+P_{rj}^{y}\geq \Delta y_{rj}u_{r},\;\forall j,r\\ & Z_{j}+P_{ij}^{x}\geq \Delta x_{ij}v_{i},\;\forall j,i\\ & Z_{0},Z_{j},P_{r0}^{y},P_{rj}^{y},P_{i0}^{x},P_{ij}^{x},u_{r},v_{i}\geq 0,\;\forall j,r,i \end{array}$$ (26)

Note that in this step, six robust data envelopment analysis (RDEA) models that are popular in the DEA field are proposed.

#### Step 1.5. Run the RDEA model for desired Γ and Δ

In the fifth step of phase 1, the robust DEA model with consideration of the conservatism level Γ and perturbation Δ for performance measurement of all stocks will be run. Also, by applying the RDEA Model, all stocks will be ranked. For the constraint *i* to be violated with probability at most *δ*_*i*_, it is sufficient to choose Γ_*i*_ at least equal to Eq (27):

$$1-\delta_{i}=1-\Phi (\frac{\Gamma_{i}-1}{\sqrt{n}})\;\Leftrightarrow \Gamma_{i}=1+\Phi_{\left(1-\delta_{i}\right)}^{-1}\sqrt{n}$$ (27)

Where Φ, the cumulative distribution, is function of the standard Gaussian variable and *n* is the number of uncertain parameters in the constraint *i*.

#### Step 1.6. Selection of top stocks from first phase

In the sixth step of phase 1, with respect to cardinality constraint ∑*τ*_*j*_ = *k* for portfolio selection in the second phase, top *k* stocks that qualified for pass the first phase to second phase will be selected. For conservative perspective to selection of the best stocks in first phase, top *k* stocks will be selected based on the average rank of per stock in all RDEA models contain of RCCR-IO, RCCR-OO, RBCC-IO, RBCC-OO, RADD-CRS and RADD-VRS models.

### 4.2. Phase II: Portfolio optimization

In this phase with 5 steps, the amount to be invested in each qualified stock is decided and finally the portfolio is created. In other words, in this phase DM makes a decision for weights of qualified stocks from the first phase in the portfolio.

#### Step 2.1. Proposing the portfolio optimization (PS) model for qualified stocks

In the first step of phase 2, two portfolio optimization models with consideration of risk, return and liquidity will be proposed. In the first model, semi variance and in the second model, absolute deviation are risk measures, respectively. For consideration of return and liquidity, two constraints are added to each model that ensures achieving the desired minimum expected return and desired minimum expected liquidity of investor. Also, in order to develop the model for covering the financial market constraint, cardinality constraint and purchasing limitation should be considered.

Now, the mean-semi variance-liquidity (MSVL) model and the mean-absolute deviation-liquidity (MADL) model are proposed as Models (29) and (30), respectively:

$$\begin{array}{ll} & \Theta^{\text{MSVL}}\\ \text{Min} & \frac{1}{\text{T}}\sum_{t=1}^{T}\xi_{t}^{2}\\ \text{S}.\text{t}. & \sum_{j=1}^{n}{\bar{R}}_{j}\omega_{j}\geq R_{E}\\ & \sum_{j=1}^{n}{\bar{L}}_{j}\omega_{j}\geq L_{E}\\ & \xi_{t}\geq R_{E}-\sum_{j=1}^{n}R_{tj}\omega_{j},\;\forall t\\ & \;\sum_{j=1}^{n}\omega_{j}=1\\ & \sum_{j=1}^{n}\tau_{j}=k\\ & A_{j}\tau_{j}\leq \omega_{j}\leq B_{j}\tau_{j},\;\forall j\\ & \tau_{j}\in \{0,1\},\;\forall j\\ & \xi_{t},\omega_{j}\geq 0,\;\forall t,j \end{array}$$ (28)

$$\begin{array}{ll} & \Theta^{\text{MADL}}\\ \text{Min} & \frac{1}{\text{T}}\sum_{t=1}^{T}\zeta_{t}\\ \text{S}.\text{t}. & \sum_{j=1}^{n}{\bar{R}}_{j}\omega_{j}\geq R_{E}\\ & \sum_{j=1}^{n}{\bar{L}}_{j}\omega_{j}\geq L_{E}\\ & \zeta_{t}\geq R_{E}-\sum_{j=1}^{n}R_{tj}\omega_{j},\;\forall t\\ & \zeta_{t}\geq \sum_{j=1}^{n}R_{tj}\omega_{j}-R_{E},\;\forall t\\ & \sum_{j=1}^{n}\omega_{j}=1\\ & \sum_{j=1}^{n}\tau_{j}=k\\ & A_{j}\tau_{j}\leq \omega_{j}\leq B_{j}\tau_{j},\;\forall j\\ & \tau_{j}\in \{0,1\},\;\forall j\\ & \zeta_{t},\omega_{j}\geq 0,\;\forall t,j \end{array}$$ (29)

It is worth noting that in MSVL and MADL models, cardinality constraint ∑*τ*_*j*_ = *k* for portfolio selection is satisfied by first phase.

#### Step 2.2. Choose a robust optimization approach

In the second step of phase 2, the Bertsimas & Sim’s [110] robust approach is selected for dealing to uncertain data and parameters in MSVL and MADL models. It should be noted that the formulation of robust counterpart in the B&S robust approach is presented in the Subsection 3.4.

#### Step 2.3. Proposing robust Portfolio Optimization (RPO) models

In the third step of phase 2, robust portfolio optimization models will be proposed. This step is the most important step in the second phase. According to B&S robust approach, the RMSVL and RMADL models are proposed as Models (30) and (31):

$$\begin{array}{ll} & \Theta_{\text{Robust}}^{\text{MSVL}}\\ \text{Min} & \frac{1}{\text{T}}\sum_{t=1}^{T}\xi_{t}^{2}\\ \text{S}.\text{t}. & -\sum_{j=1}^{n}{\bar{R}}_{j}\omega_{j}+Z^{\bar{R}}\Gamma^{\bar{R}}+\sum_{j=1}^{n}P_{j}^{\bar{R}}\leq -R_{E}\\ & -\sum_{j=1}^{n}{\bar{L}}_{j}\omega_{j}+Z^{\bar{L}}\Gamma^{\bar{L}}+\sum_{j=1}^{n}P_{j}^{\bar{L}}\leq -L_{E}\\ & R_{E}-\sum_{j=1}^{n}R_{tj}\omega_{j}+Z_{j}^{R}\Gamma_{j}^{R}+\sum_{j=1}^{n}P_{tj}^{R}\leq \xi_{t},\;\forall t,j\\ & \sum_{j=1}^{n}\omega_{j}=1\\ & \sum_{j=1}^{n}\tau_{j}=k\\ & Z^{\bar{R}}+P_{j}^{\bar{R}}\geq \Delta {\bar{R}}_{j}\omega_{j},\;\forall j\\ & Z^{\bar{L}}+P_{j}^{\bar{L}}\geq \Delta {\bar{L}}_{j}\omega_{j},\;\forall j\\ & Z_{j}^{R}+P_{tj}^{R}\geq \Delta R_{tj}\omega_{j},\;\forall t,j\\ & Z^{\bar{R}},Z^{\bar{L}},Z_{j}^{R},P_{j}^{\bar{R}},P_{j}^{\bar{L}},P_{tj}^{R}\geq 0,\;\forall t,j\\ & A_{j}\tau_{j}\leq \omega_{j}\leq B_{j}\tau_{j},\;\forall j\\ & \tau_{j}\in \{0,1\},\;\forall j\\ & \xi_{t},\omega_{j}\geq 0,\;\forall t,j \end{array}$$ (30)

$$\begin{array}{ll} & \Theta_{\text{Robust}}^{\text{MADL}}\\ \text{Min} & \frac{1}{\text{T}}\sum_{t=1}^{T}\zeta_{t}\\ \text{S}.\text{t}. & -\sum_{j=1}^{n}{\bar{R}}_{j}\omega_{j}+Z^{\bar{R}}\Gamma^{\bar{R}}+\sum_{j=1}^{n}P_{j}^{\bar{R}}\leq -R_{E}\\ & -\sum_{j=1}^{n}{\bar{L}}_{j}\omega_{j}+Z^{\bar{L}}\Gamma^{\bar{L}}+\sum_{j=1}^{n}P_{j}^{\bar{L}}\leq -L_{E}\\ & R_{E}-\sum_{j=1}^{n}R_{tj}\omega_{j}+Z_{j}^{R_{1}}\Gamma_{j}^{R_{1}}+\sum_{j=1}^{n}P_{tj}^{R_{1}}\leq \zeta_{t},\;\forall t,j\\ & -R_{E}+\sum_{j=1}^{n}R_{tj}\omega_{j}+Z_{j}^{R_{2}}\Gamma_{j}^{R_{2}}+\sum_{j=1}^{n}P_{tj}^{R_{2}}\leq \zeta_{t},\;\forall t,j\\ & \sum_{j=1}^{n}\omega_{j}=1\\ & \sum_{j=1}^{n}\tau_{j}=k\\ & Z^{\bar{R}}+P_{j}^{\bar{R}}\geq \Delta {\bar{R}}_{j}\omega_{j},\;\forall j\\ & Z^{\bar{L}}+P_{j}^{\bar{L}}\geq \Delta {\bar{L}}_{j}\omega_{j},\;\forall j\\ & Z_{j}^{R_{1}}+P_{tj}^{R_{1}}\geq \Delta R_{tj}\omega_{j},\;\forall t,j\\ & Z_{j}^{R_{2}}+P_{tj}^{R_{2}}\geq \Delta R_{tj}\omega_{j},\;\forall t,j\\ & Z^{\bar{R}},Z^{\bar{L}},Z_{j}^{R_{1}},Z_{j}^{R_{2}},P_{j}^{\bar{R}},P_{j}^{\bar{L}},P_{tj}^{R_{1}},P_{tj}^{R_{2}}\geq 0,\;\forall t,j\\ & A_{j}\tau_{j}\leq \omega_{j}\leq B_{j}\tau_{j},\;\forall j\\ & \tau_{j}\in \{0,1\},\;\forall j\\ & \zeta_{t},\omega_{j}\geq 0,\;\forall t,j \end{array}$$ (31)

In this step, two robust portfolio optimization models that are RMSVL and RMADL are proposed.

#### Step 2.4. Run the RPS model to achieve desired Γ and Δ

In the fourth step of phase 2, the robust portfolio optimization model with consideration of the desired level of conservatism Γ and perturbation Δ is run to make a decision for weights of the qualified stocks obtained from the first phase. As same as the fifth step of phase 1, for the constraint *i* to be violated with probability at most *δ*_*i*_, it is sufficient to choose Γ_*i*_ at least equal to Eq (27).

#### Step 2.5. Portfolio construction with weights of the RPO model

In the fifth step of phase 2, finally, with respect to weights of top *k* stocks in the RMSVL and RMADL models, the investor desired portfolio will be constructed. It should be noted that, with changing the desired minimum expected return and desired minimum expected liquidity of the investor, the efficient frontier will be made.

## 5. Case study and numerical results

In this section, the implementation of the proposed approach of this paper for the portfolio construction problem, is presented for a real-world case study from Tehran stock exchange (TSE). TSE, with a history of nearly half a century, is one of the most attractive financial markets in the Middle East region. Pharmaceutical industry involving 27 stocks is selected and financial data are extracted from March 2013 to March 2014. Summary of real-world data from Pharmaceutical industry of Tehran stock exchange (TSE) that are used in this research are as Table 4.

**Table 4:** Summary of real-world data from Tehran Stock Exchange (TSE).

Stocks	Inputs					Outputs			
	I (1)	I (2)	I (3)	I (4)	I (5)	O (1)	O (2)	O (3)	O (4)
PDRO	7.43	1.18	1.22	1.03	0.02	3344	1.93	157.67	59.33
DLGM	13.38	0.49	3.87	0.70	0.03	213	2.06	183.48	133.33
THSH	11.58	0.59	2.85	0.01	0.02	799	0.69	110.28	30.16
DDPK	7.70	0.86	2.27	0.54	0.05	693	2.73	122.76	56.85
TMVD	6.58	1.16	1.00	0.64	0.02	2965	1.04	166.99	10.66
DAML	8.70	0.87	3.91	0.57	0.03	1386	1.98	156.08	2.74
DFRB	7.76	1.07	1.84	1.40	0.03	1277	2.04	164.07	31.17
DKSR	8.96	0.97	1.36	1.48	0.03	121	2.64	228.88	369.42
DARO	7.93	7.07	0.10	1.27	0.03	1553	1.85	187.63	54.67
DABO	9.03	0.86	3.44	0.71	0.03	1357	2.30	143.68	93.15
DRZK	7.91	0.96	1.72	0.68	0.03	1493	2.88	167.43	96.65
DOSE	18.43	1.06	1.23	1.56	0.04	997	1.92	169.70	67.00
PKSH	6.41	0.90	5.95	1.67	0.03	528	0.73	227.86	53.22
IRDR	7.47	0.72	3.00	1.09	0.03	306	1.59	187.99	230.39
DALZ	7.46	1.28	1.21	1.49	0.03	956	2.49	205.22	111.30
DSBH	8.39	1.35	0.86	1.60	0.04	2340	2.91	155.82	95.56
DPAK	6.82	0.79	4.43	1.30	0.05	666	2.52	177.08	119.82
DJBR	6.94	1.21	0.94	0.94	0.03	659	3.14	219.36	122.76
KIMI	6.81	0.73	2.28	6.24	0.21	227	5.74	147.27	438.33
EXIR	8.20	0.82	5.16	1.14	0.03	1283	3.14	198.36	118.24
DSIN	7.52	1.21	0.84	0.97	0.03	1222	1.80	174.39	94.68
ROZD	8.84	1.01	0.95	0.28	0.07	131	1.46	26.37	286.26
AMIN	5.73	0.97	1.45	0.74	0.04	696	4.15	163.71	230.03
DZAH	5.40	0.95	2.83	1.20	0.07	2699	2.35	44.51	129.27
ABDI	10.22	0.60	4.81	0.59	0.03	404	2.21	181.41	83.42
ALBZ	6.90	1.00	1.93	1.41	0.03	418	1.49	228.42	104.07
DSOB	6.75	1.06	1.57	1.46	0.03	655	2.65	221.73	104.58
Mean	8.34	1.18	2.33	1.21	0.04	1088.44	2.31	167.34	123.22
SD	2.58	1.17	1.50	1.07	0.04	851.51	1.03	48.01	101.97
Max	18.43	7.07	5.95	6.24	0.21	3344	5.74	228.88	438.33
Min	5.40	0.49	0.10	0.01	0.02	121	0.69	26.37	2.74

Now, after collecting data, the robust CCR-IO, robust CCR-OO, robust BCC-IO, robust BCC-OO, robust ADD-CRS and robust ADD-VRS models will be run. According to the desired confidence level of 90% in order to satisfy the constraints in the robust data envelopment analysis models, based on Eq (27), the level of conservatism Γ is set equal to 3.56, 3.86 and 4.84 for constraints with 4, 5 and 9 uncertain parameters, respectively. Also, the perturbations Δ is set to 0.05. The results of all RDEA models that are presented in Model (21) to (26) are introduced in Table 5.

**Table 5:** The results of robust DEA models.

Stocks	Robust CCR-IO	Robust CCR-OO	Robust BCC-IO	Robust BCC-OO	Robust ADD-CRS	Robust ADD-VRS
PDRO	0.85887	1.16433	0.88791	1.10312	29.87672	22.87556
DLGM	0.84602	1.18200	0.90960	1.13212	25.70614	22.84913
THSH	0.86882	1.15099	0.91623	1.10438	5.15744	4.06436
DDPK	0.71995	1.38898	0.88951	1.34676	61.67257	55.00222
TMVD	0.85481	1.16984	0.91623	1.10534	25.36754	22.85278
DAML	0.79114	1.26399	0.90124	1.21064	39.54300	35.12576
DFRB	0.69491	1.43904	0.84832	1.34537	76.09811	62.20772
DKSR	0.85595	1.16830	0.90727	1.10470	32.45019	22.84938
DARO	0.85575	1.16856	0.91623	1.10595	26.44291	19.18050
DABO	0.76187	1.31256	0.87295	1.30348	51.04647	48.93671
DRZK	0.85135	1.17460	0.90870	1.12988	27.15691	23.33055
DOSE	0.67946	1.47177	0.87701	1.35464	86.38817	62.94803
PKSH	0.82837	1.20720	0.90559	1.10516	38.97710	22.91794
IRDR	0.83047	1.20414	0.90969	1.18742	34.87790	32.73420
DALZ	0.73721	1.35646	0.80370	1.14626	65.60089	30.61510
DSBH	0.85606	1.16814	0.90077	1.10779	32.14735	24.66641
DPAK	0.79327	1.26060	0.90483	1.25902	44.12418	44.11159
DJBR	0.85682	1.16711	0.90937	1.10447	27.74941	22.73128
KIMI	0.84950	1.17716	0.90722	1.10386	34.44683	23.76253
EXIR	0.84526	1.18306	0.89649	1.10443	33.07610	23.57070
DSIN	0.80445	1.24309	0.91155	1.24237	38.35772	36.18096
ROZD	0.84649	1.18135	0.91623	1.11277	16.50960	16.31644
AMIN	0.86319	1.15850	0.91174	1.10522	25.74536	22.44055
DZAH	0.84416	1.18461	0.90703	1.10841	40.36772	31.02423
ABDI	0.84234	1.18717	0.90652	1.13665	27.02603	22.44868
ALBZ	0.83444	1.19841	0.90530	1.10507	38.56758	22.87104
DSOB	0.83244	1.20129	0.87665	1.10501	39.79929	22.87686

After running all RDEA models, the ranking of all stocks in RCCR-IO, RCCR-OO, RBCC-IO, RBCC-OO, RADD-CRS and RADD-VRS models are presented in Table 6.

**Table 6:** The ranking of stocks in robust DEA models.

Stocks	Robust CCR-IO	Robust CCR-OO	Robust BCC-IO	Robust BCC-OO	Robust ADD-CRS	Robust ADD-VRS	Average
PDRO	3	3	22	1	10	11	**8**
DLGM	12	12	8	17	4	7	**9**
THSH	1	1	1	3	1	1	**1**
DDPK	25	25	21	26	24	25	25
TMVD	8	8	1	11	3	9	**5**
DAML	22	22	18	21	19	21	21
DFRB	26	26	26	25	26	26	26
DKSR	6	6	11	6	12	8	**7**
DARO	7	7	1	12	6	3	**3**
DABO	23	23	25	24	23	24	24
DRZK	9	9	10	16	8	14	11
DOSE	27	27	23	27	27	27	27
PKSH	19	19	15	9	18	13	16
IRDR	18	18	7	20	15	20	19
DALZ	24	24	27	19	25	18	23
DSBH	5	5	19	13	11	17	12
DPAK	21	21	17	23	22	23	22
DJBR	4	4	9	5	9	6	**4**
KIMI	10	10	12	2	14	16	**10**
EXIR	13	13	20	4	13	15	14
DSIN	20	20	6	22	16	22	20
ROZD	11	11	1	15	2	2	**6**
AMIN	2	2	5	10	5	4	**2**
DZAH	14	14	13	14	21	19	17
ABDI	15	15	14	18	7	5	13
ALBZ	16	16	16	8	17	10	15
DSOB	17	17	24	7	20	12	18

According to cardinality constraint in RMSVL and RMADL, *k* is set equal to 10, ten stocks that have a higher average rank in Table 6 are selected. Finally, the set of stocks that selected from RDEA models are PDRO, DLGM, THSH, TMVD, DKSR, DARO, DJBR, KIMI, ROZD, and AMIN. In order to run RMSVL and RMADL models, the monthly data for the return and the liquidity of the selected stocks are extracted for 12 months between March 2013 to March 2014 from TSE. The real data for the return and the liquidity of the selected stocks per 12 periods are presented in Tables 7 and 8, respectively:

**Table 7:** The return of stocks per period.

Periods	Selected Stocks in Phase 1									
	PDRO	DLGM	THSH	TMVD	DKSR	DARO	DJBR	KIMI	ROZD	AMIN
Period 1^th^	0.0160	0.2266	-0.0031	0.1359	0.1685	0.0097	0.0508	0.2122	0.0071	0.1094
Period 2^th^	-0.1193	-0.0638	-0.2154	0.0289	0.0012	0.0563	0.1100	0.5835	0.0049	0.1238
Period 3^th^	0.3156	0.1300	0.1015	0.3195	0.3108	0.0468	0.3073	0.6966	0.0497	0.1197
Period 4^th^	0.2472	0.4190	0.1030	0.1672	0.5002	0.7275	0.5629	0.5675	0.0829	0.0322
Period 5^th^	0.1063	0.1474	0.0232	-0.1552	-0.0924	0.0633	-0.0493	-0.2953	0.0188	0.1704
Period 6^th^	0.0909	-0.1696	0.1883	0.0055	0.0133	0.1084	0.0361	-0.0666	0.0028	0.3193
Period 7^th^	0.2347	0.1540	0.0636	0.1367	0.3673	0.0068	0.1900	-0.0393	0.0118	0.3822
Period 8^th^	0.3047	0.3167	0.0343	0.1907	0.3148	0.0769	0.4905	1.2232	0.0005	0.5867
Period 9^th^	0.1981	0.1549	0.0480	0.0689	0.2201	0.2104	0.2029	0.5297	0.1466	0.1842
Period 10^th^	-0.1011	0.0860	0.0240	-0.0097	0.0317	0.2085	-0.1096	-0.0594	0.1328	-0.0580
Period 11^th^	0.0028	-0.0609	0.2036	-0.0174	-0.2285	-0.1966	-0.1507	-0.1246	0.0161	0.0803
Period 12^th^	0.0483	-0.0157	0.0332	-0.0210	0.0523	0.0066	0.2050	-0.0113	0.5774	-0.0563
Average Return	0.1120	0.1104	0.0504	0.0708	0.1383	0.1104	0.1538	0.2680	0.0876	0.1662

**Table 8:** The liquidity of stocks per period.

Periods	Selected Stocks in Phase 1									
	PDRO	DLGM	THSH	TMVD	DKSR	DARO	DJBR	KIMI	ROZD	AMIN
Period 1^th^	9.83	14.76	3.91	8.41	14.2	6.62	12.19	14.07	1.20	10.67
Period 2^th^	18.08	11.97	14.55	16.38	21.04	19.55	19.43	5.31	1.33	14.79
Period 3^th^	17.78	1.81	15.79	14.24	18.37	8.69	12.33	7.35	3.51	7.01
Period 4^th^	10.52	14.66	16.47	20.09	18.36	15.84	20.57	11.23	3.34	4.62
Period 5^th^	15.96	15.00	9.43	18.88	18.54	18.10	19.31	19.15	3.41	5.21
Period 6^th^	18.77	14.35	12.73	18.55	19.72	14.76	19.79	20.07	1.00	17.48
Period 7^th^	19.10	18.94	17.66	19.03	20.52	18.52	19.59	5.76	1.20	9.58
Period 8^th^	15.99	18.60	9.09	18.89	19.69	17.33	19.59	4.97	0.67	12.88
Period 9^th^	20.10	19.80	9.34	20.74	20.78	14.80	20.65	13.87	0.97	20.47
Period 10^th^	17.74	15.31	6.29	18.26	18.77	18.05	14.78	18.8	1.55	18.20
Period 11^th^	13.63	18.84	12.82	15.49	17.25	20.07	20.31	14.31	4.03	17.05
Period 12^th^	14.23	18.00	8.44	17.98	19.39	19.11	19.25	16.73	2.70	18.92
Average Liquidity	15.98	15.17	11.38	17.25	18.89	15.95	18.15	12.64	2.08	13.07

Now, after selecting stocks from the first phase, in the second phase, the robust mean-semi variance-liquidity (RMSVL) and robust mean-absolute deviation-liquidity (RMADL) models will be run. According to the desired confidence level of 90% in order to satisfy the constraints in the RMSVL and RMADL models, based on Eq (27), the level of conservatism Γ is set equal to 5.05 for a constraint with 10 uncertain parameters. Also, the perturbations Δ is set to 0.05 and taking into account the expected liquidity of portfolio is fixed equal to 10.50, and the expected return of the portfolio is increased. With considering the different expected returns of the portfolio, the results of RMSVL and RMADL models that are presented in Models (30) and (31) are introduced in Tables 9 and 10:

**Table 9:** The Results of robust Mean-Semi Variance-Liquidity (RMSVL) model.

Expected Liquidity of Portfolio					10.50			
Expected Return of Portfolio		0.060	0.090	0.120	0.150	0.180	0.210	0.240
Weight of Selected Stocks from Phase .1 in Portfolio	PDRO	0.01000	0.01000	0.01000	0.01000	0.01000	0.01000	0.01000
	DLGM	0.01000	0.03768	0.12088	0.01993	0.01000	0.01000	0.01000
	THSH	0.18201	0.08600	0.01000	0.01000	0.01000	0.01000	0.01000
	TMVD	0.01000	0.01000	0.01000	0.01000	0.01000	0.01000	0.01000
	DKSR	0.01000	0.01000	0.01000	0.01000	0.01000	0.01000	0.01000
	DARO	0.11863	0.08240	0.03309	0.01000	0.01000	0.01000	0.01000
	DJBR	0.01000	0.01000	0.01000	0.01000	0.01000	0.01000	0.01000
	KIMI	0.05947	0.03302	0.02084	0.11980	0.31677	0.58535	0.89555
	ROZD	0.20666	0.21947	0.23321	0.20386	0.06407	0.01000	0.01000
	AMIN	0.38323	0.50142	0.54198	0.59641	0.54916	0.33465	0.02445
Risk (SV) of Portfolio		0.00019	0.00086	0.00211	0.00489	0.01289	0.02839	0.05780

**Table 10:** The Results of robust Mean-Absolute Deviation-Liquidity (RMADL) model.

Expected Liquidity of Portfolio					10.50			
Expected Return of Portfolio		0.080	0.105	0.130	0.155	0.180	0.205	0.230
Weight of Selected Stocks from Phase .1 in Portfolio	PDRO	0.01000	0.01000	0.01000	0.01000	0.01000	0.01000	0.01000
	DLGM	0.20348	0.21193	0.17378	0.01029	0.01000	0.01000	0.01000
	THSH	0.46927	0.23438	0.01000	0.01000	0.01000	0.01000	0.01000
	TMVD	0.01000	0.01000	0.01435	0.01000	0.01000	0.01000	0.01000
	DKSR	0.01000	0.01000	0.01000	0.01000	0.01000	0.01000	0.01000
	DARO	0.03168	0.01000	0.01000	0.01000	0.01000	0.01000	0.01000
	DJBR	0.01000	0.01000	0.01292	0.01000	0.01000	0.01000	0.01000
	KIMI	0.01000	0.01000	0.02959	0.16350	0.28806	0.53365	0.79215
	ROZD	0.17849	0.19811	0.24012	0.20019	0.02690	0.01000	0.01000
	AMIN	0.06708	0.29558	0.48925	0.56602	0.61504	0.38635	0.12785
Risk (AD) of Portfolio		0.05130	0.05915	0.07289	0.10469	0.15438	0.23195	0.32218

**Fig 2:**
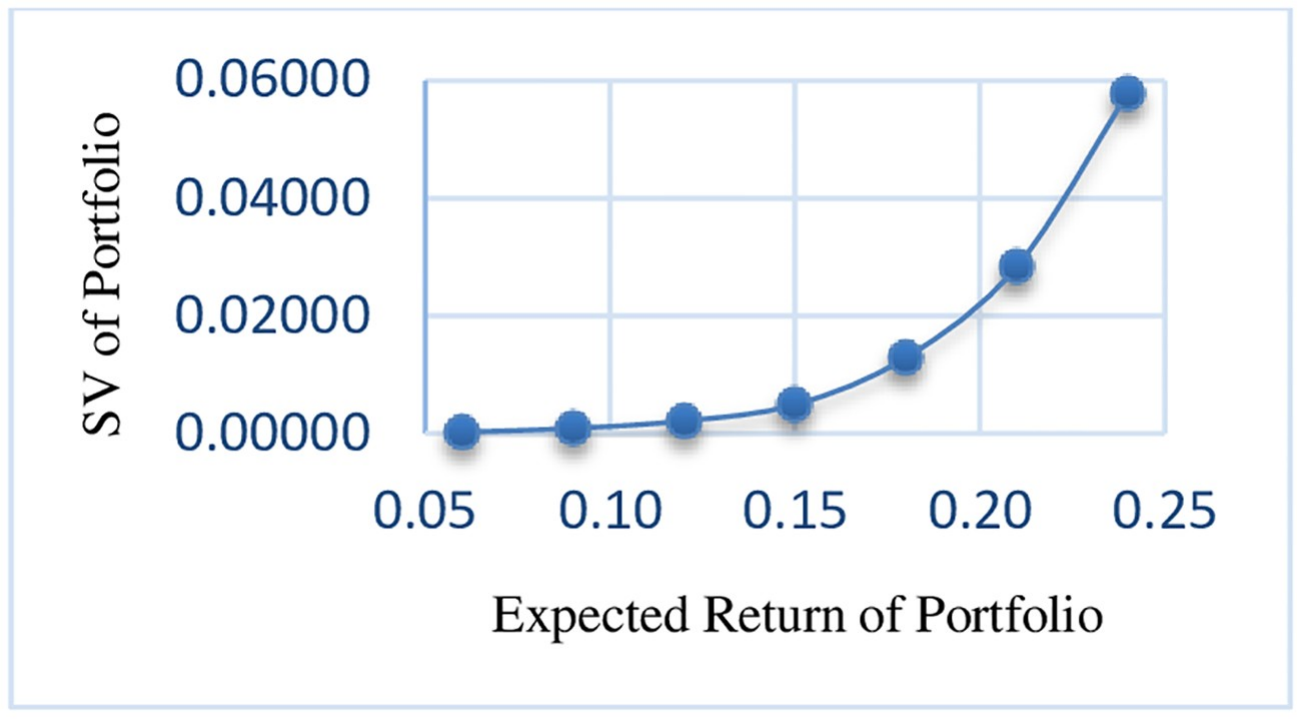
Efficient frontier of RMSVL.

**Fig 3:**
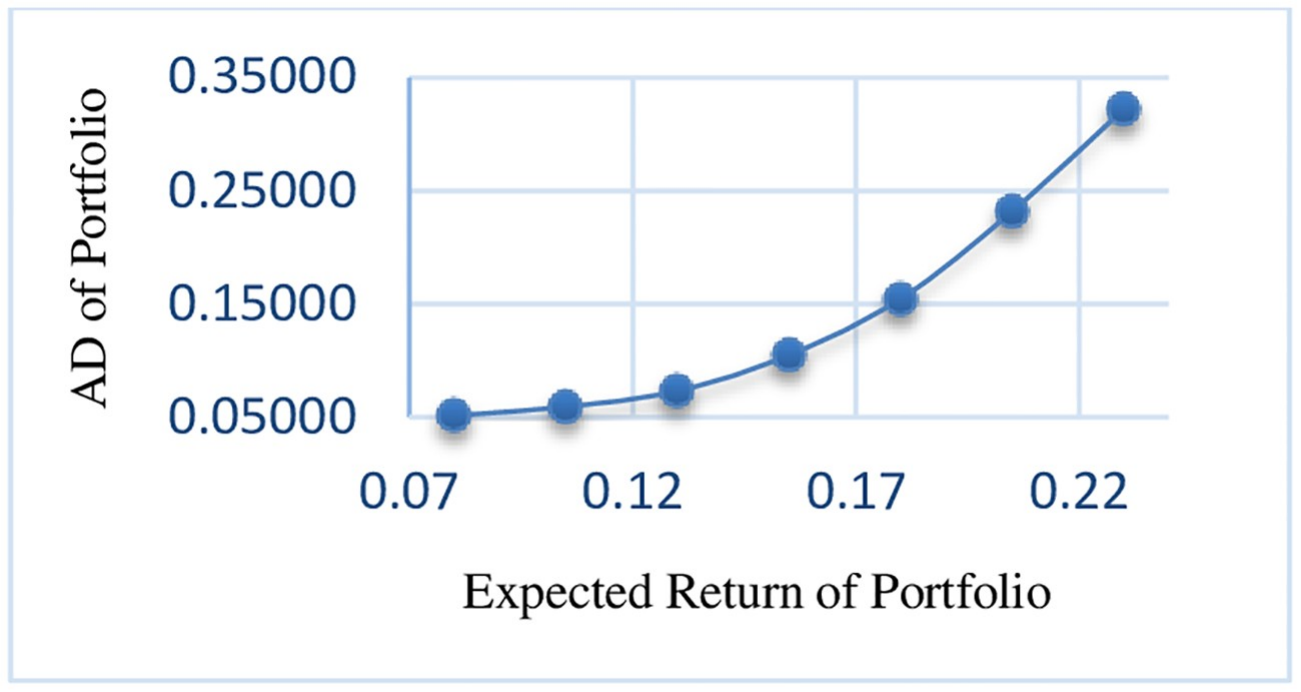
Efficient frontier of RMADL.

As can be seen in the results, with an increase in the expected return of the portfolio, the risk of portfolio is also increased. The efficient frontier of RMSVL and RMADL are presented in Figs 2 and 3, respectively.

## 6. Sensitivity analysis

**Fig 4:**
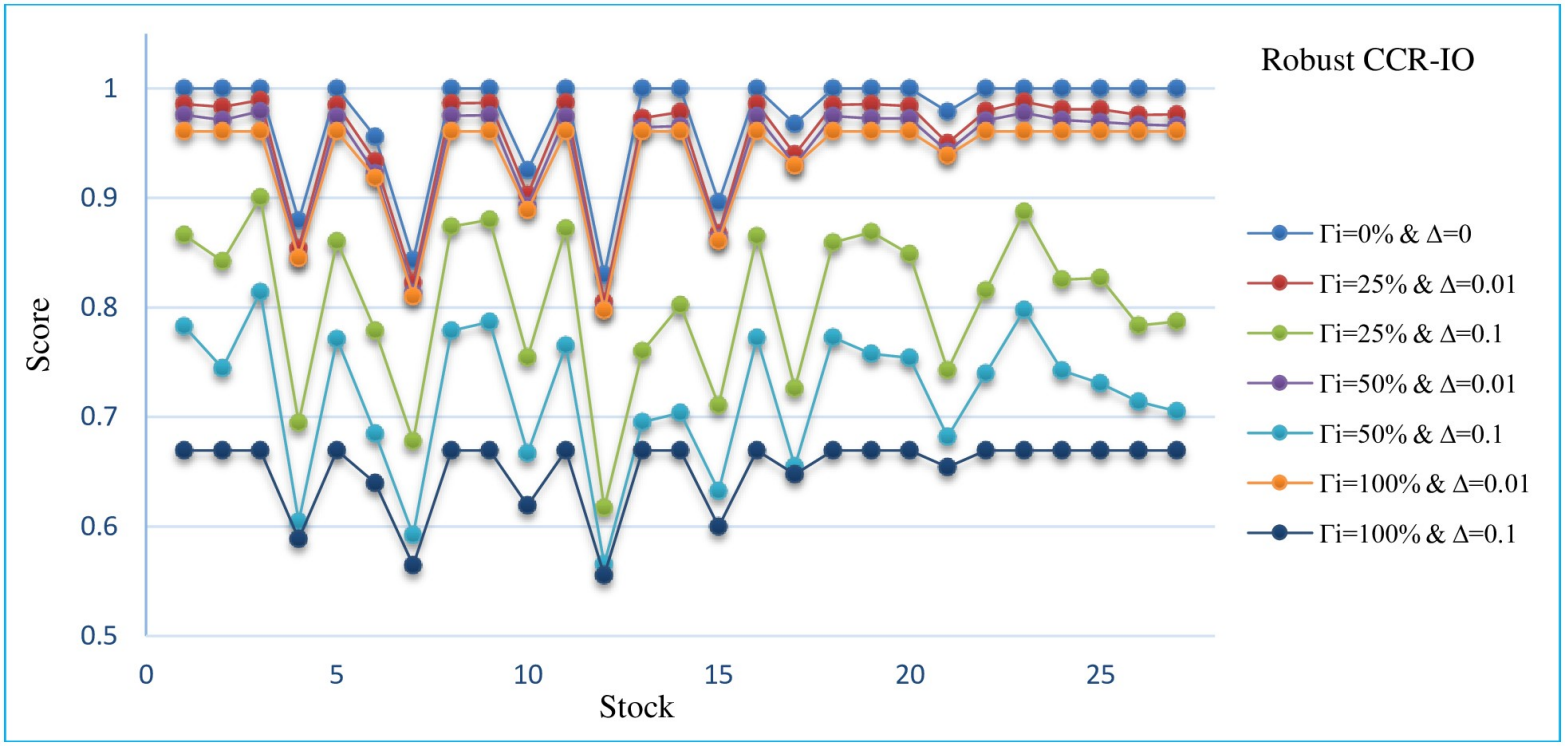
The trend of robust CCR-IO model for different Γ and Δ.

**Fig 5:**
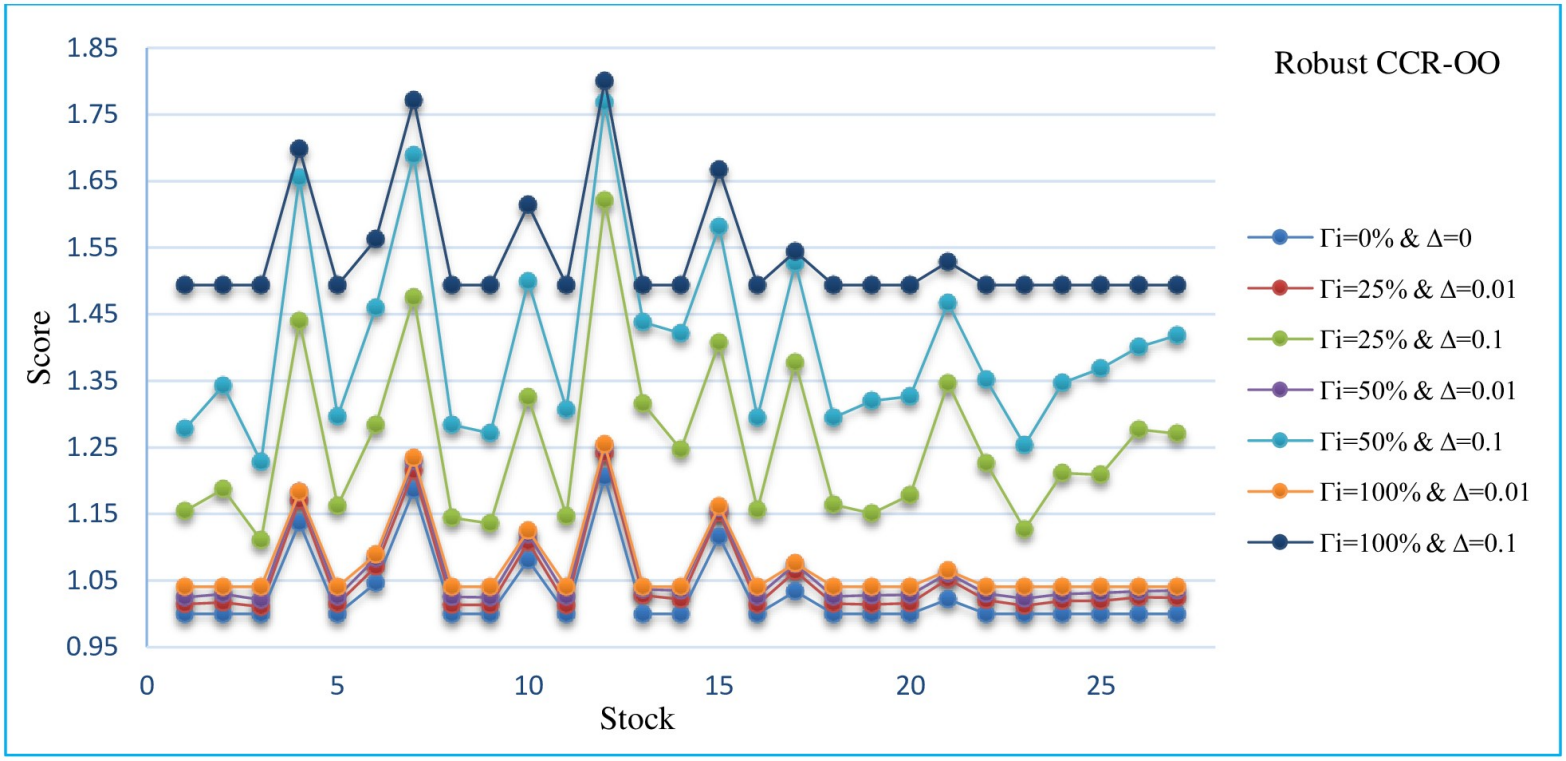
The trend of robust CCR-OO model for different Γ and Δ.

**Fig 6:**
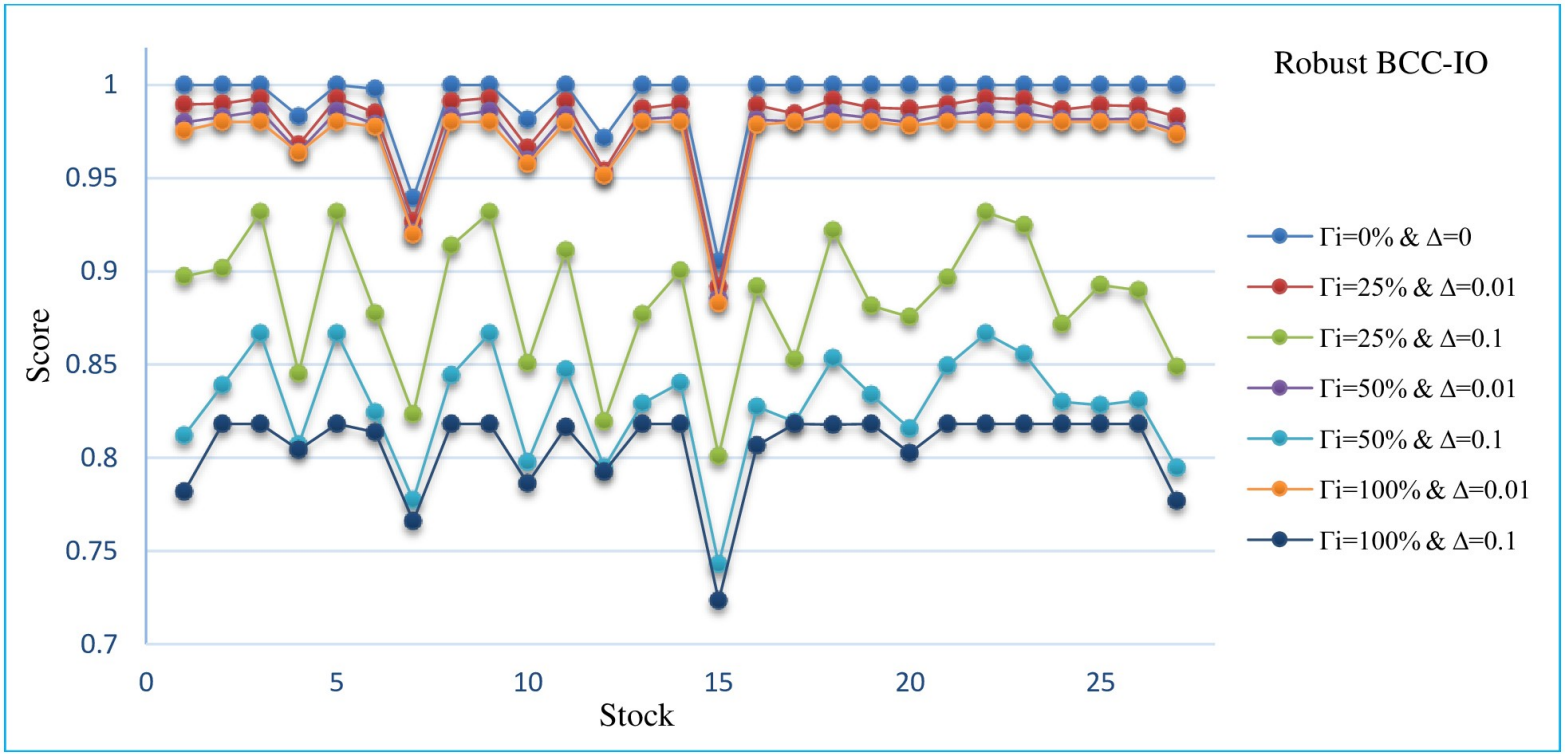
The trend of robust BCC-IO model for different Γ and Δ.

**Fig 7:**
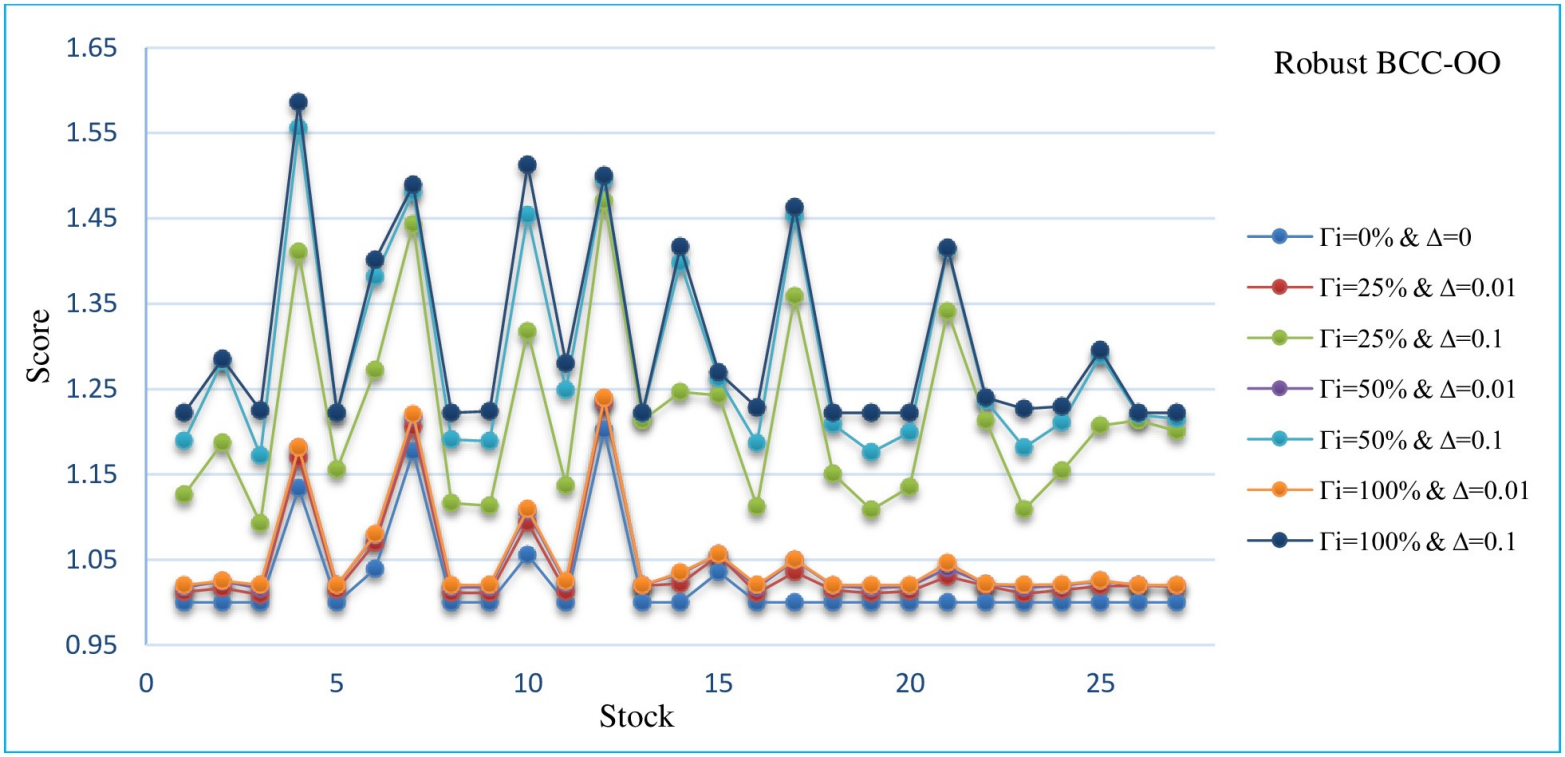
The trend of robust BCC-OO model for different Γ and Δ.

**Fig 8:**
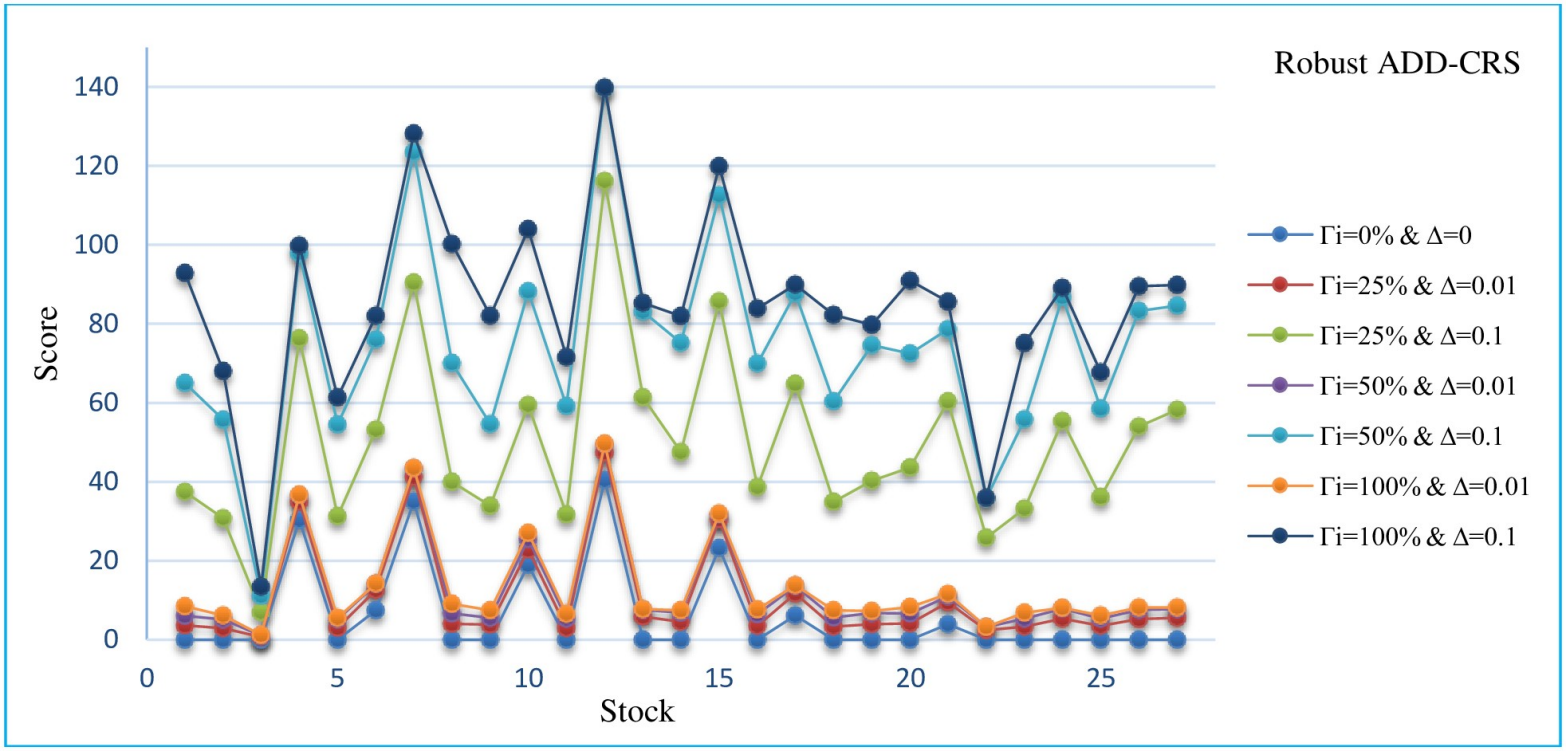
The trend of robust ADD-CRS model for different Γ and Δ.

**Fig 9:**
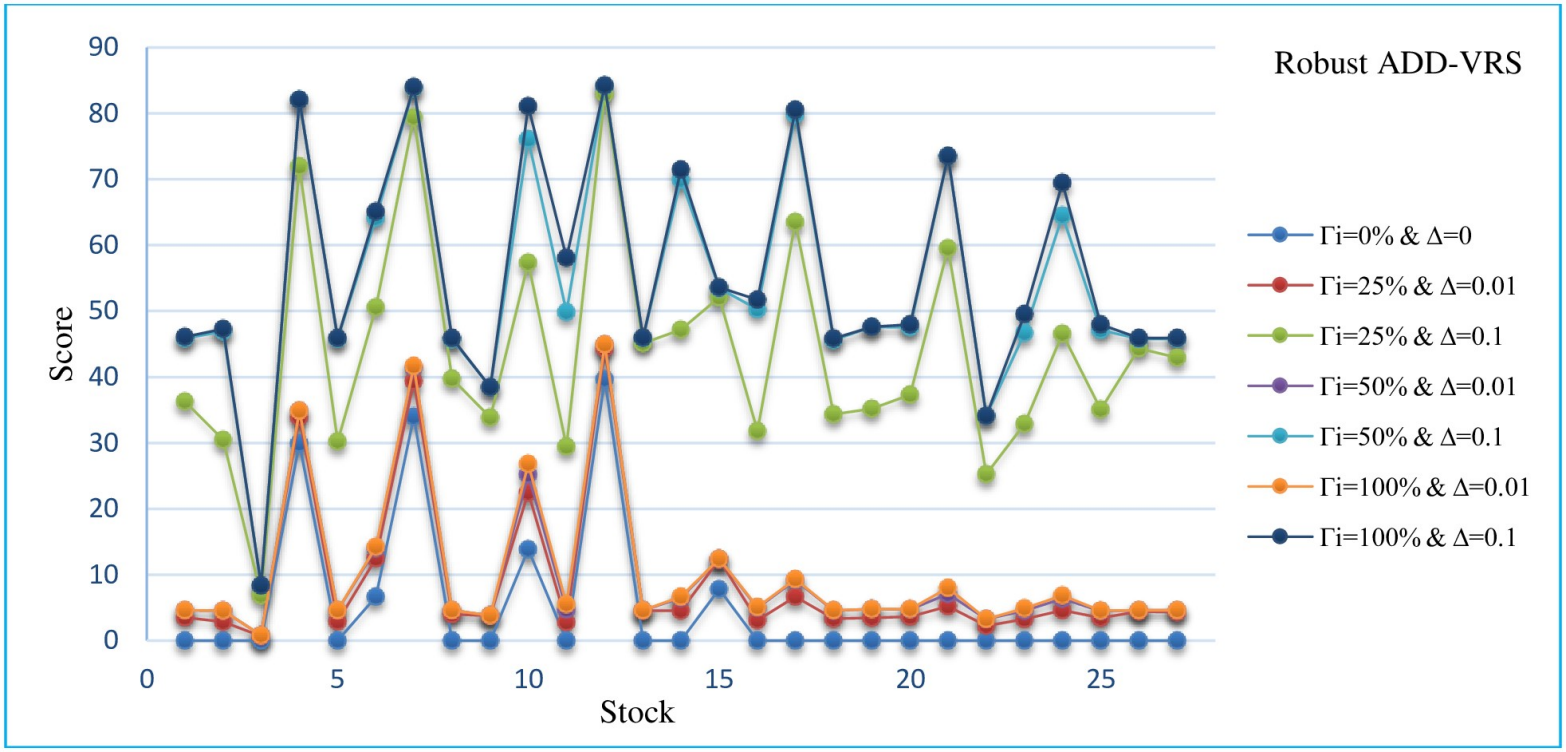
The trend of robust ADD-VRS model for different Γ and Δ.

**Fig 10:**
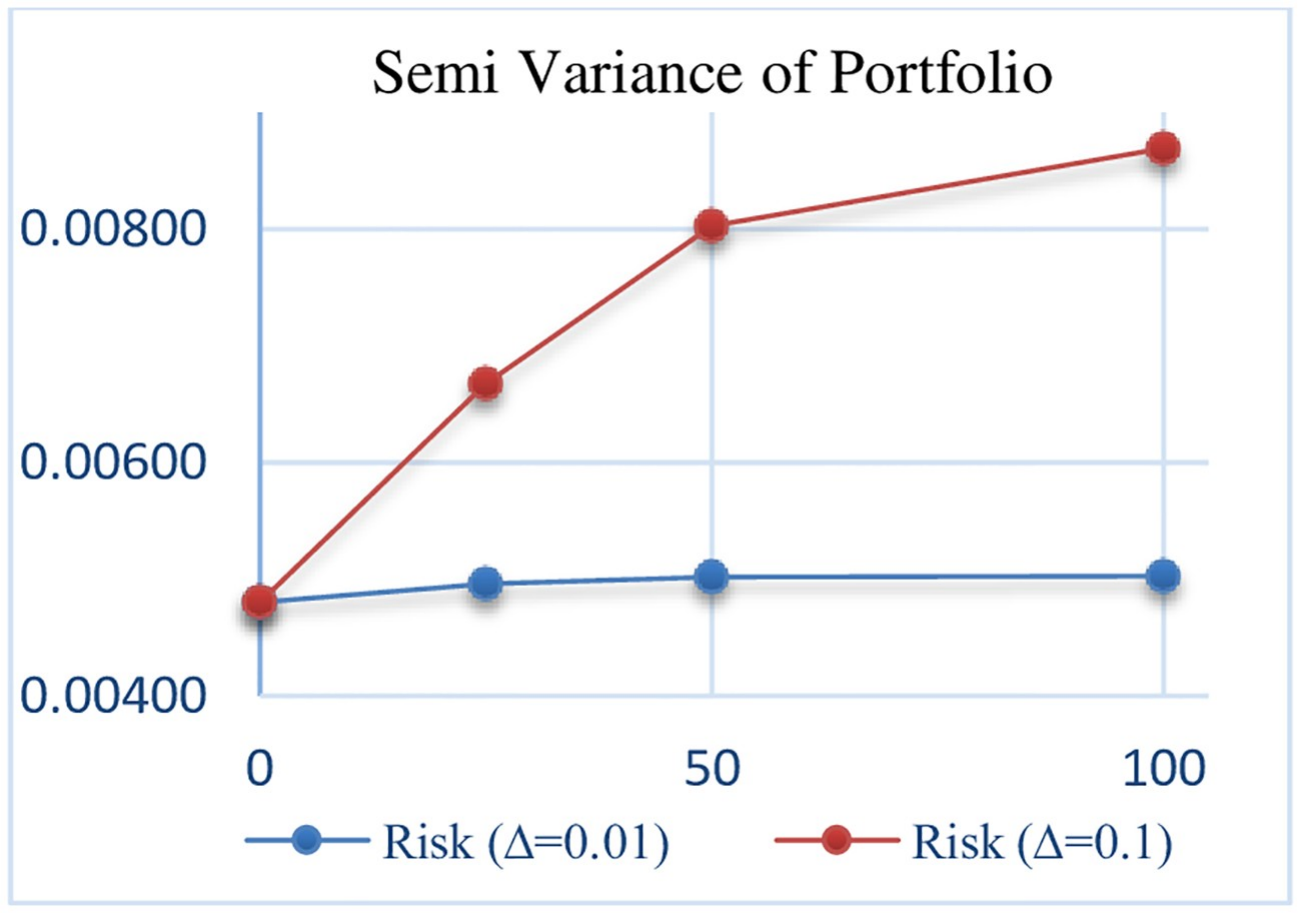
The trend of portfolio SV in RMSVL.

**Fig 11:**
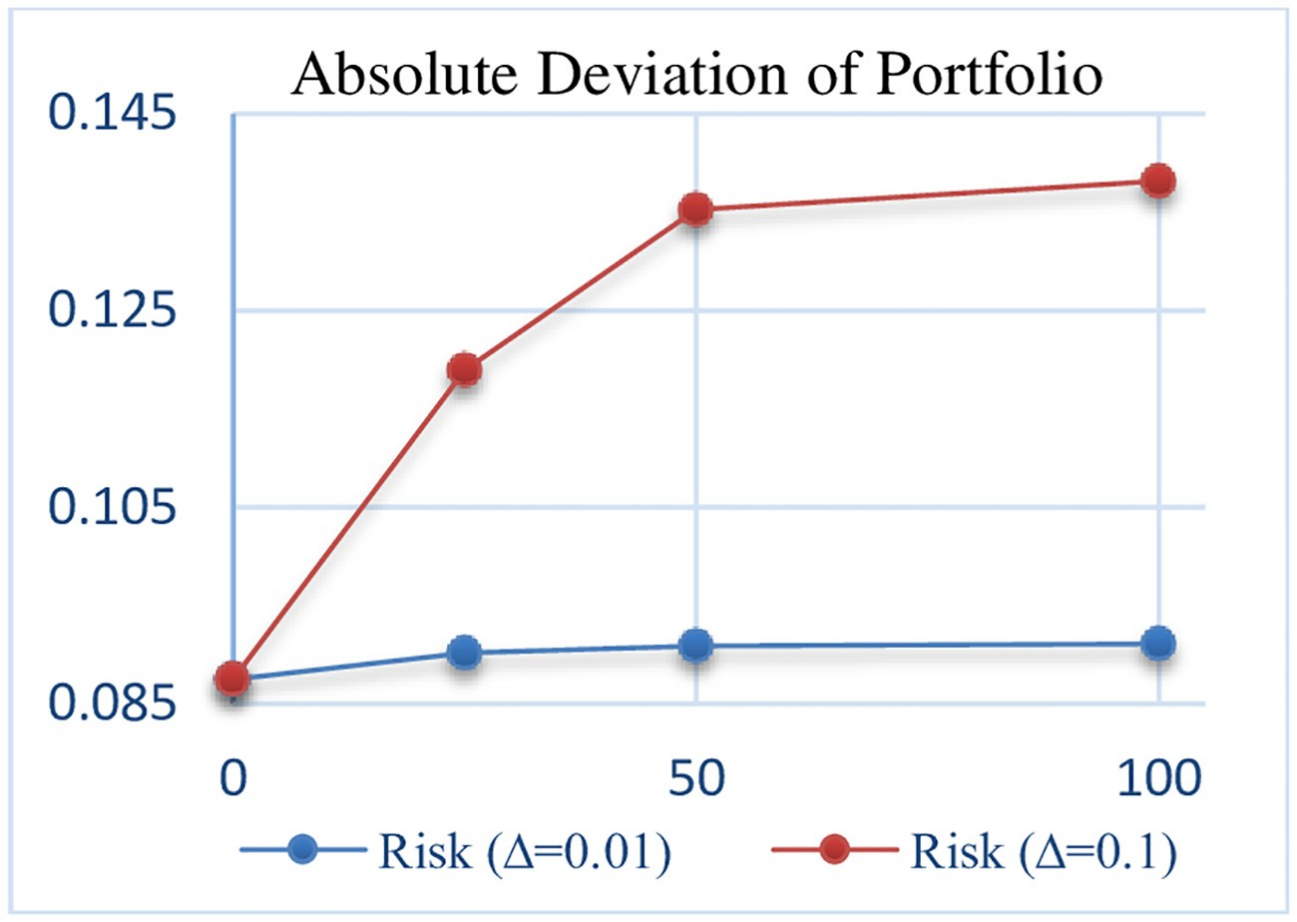
The trend of portfolio AD in RMADL.

In this section, the sensitivity analysis of all robust models that are presented for different Γand Δ. The Sensitivity analysis RCCR-IO, RCCR-OO, RBCC-IO, RBCC-OO, RADD-CRS, RADD-VRS, RMSVL and RMADL models are presented in Tables 11–18, respectively. Also, the trend of results from all robust models are introduced in Figs 4–11, respectively:

**Table 11:** The results of robust CCR-IO model with different Γ and Δ.

Stocks	CCR-IO	Robust CCR-IO					
		Γ_i_ = 25%		Γ_i_ = 50%		Γ_i_ = 100%	
		Δ = 0.01	Δ = 0.1	Δ = 0.01	Δ = 0.1	Δ = 0.01	Δ = 0.1
PDRO	1	0.98576	0.86619	0.97577	0.78265	0.96079	0.66942
DLGM	1	0.98321	0.84203	0.97131	0.74458	0.96079	0.66942
THSH	1	0.98945	0.90056	0.97927	0.81429	0.96079	0.66942
DDPK	0.87950	0.85406	0.69452	0.84501	0.60422	0.84501	0.58876
TMVD	1	0.98493	0.86045	0.97446	0.77128	0.96079	0.66942
DAML	0.95574	0.93380	0.77865	0.92271	0.68497	0.91826	0.63979
DFRB	0.84316	0.82303	0.67786	0.81337	0.59220	0.81010	0.56443
DKSR	1	0.98649	0.87389	0.97521	0.77880	0.96079	0.66942
DARO	1	0.98682	0.88025	0.97570	0.78672	0.96079	0.66942
DABO	0.92518	0.90290	0.75447	0.89289	0.66705	0.88890	0.61934
DRZK	1	0.98708	0.87209	0.97446	0.76513	0.96079	0.66942
DOSE	0.82973	0.80513	0.61687	0.79845	0.56551	0.79719	0.55544
PKSH	1	0.97310	0.76003	0.96464	0.69536	0.96079	0.66942
IRDR	1	0.97847	0.80217	0.96586	0.70360	0.96079	0.66942
DALZ	0.89608	0.86782	0.71059	0.86329	0.63237	0.86094	0.59985
DSBH	1	0.98608	0.86503	0.97475	0.77227	0.96079	0.66942
DPAK	0.96745	0.93981	0.72583	0.93040	0.65497	0.92951	0.64763
DJBR	1	0.98496	0.85913	0.97481	0.77250	0.96079	0.66942
KIMI	1	0.98591	0.86883	0.97273	0.75750	0.96079	0.66942
EXIR	1	0.98407	0.84879	0.97252	0.75397	0.96079	0.66942
DSIN	0.97871	0.95010	0.74282	0.94241	0.68175	0.93873	0.65423
ROZD	1	0.97968	0.81546	0.97065	0.73979	0.96079	0.66942
AMIN	1	0.98801	0.88741	0.97754	0.79776	0.96079	0.66942
DZAH	1	0.98112	0.82557	0.97107	0.74233	0.96079	0.66942
ABDI	1	0.98097	0.82698	0.96949	0.73059	0.96079	0.66942
ALBZ	1	0.97586	0.78337	0.96725	0.71394	0.96079	0.66942
DSOB	1	0.97630	0.78709	0.96607	0.70501	0.96079	0.66942

**Table 12:** The results of robust CCR-OO model with different Γ and Δ.

Stocks	CCR-OO	Robust CCR-OO					
		Γ_i_ = 25%		Γ_i_ = 50%		Γ_i_ = 100%	
		Δ = 0.01	Δ = 0.1	Δ = 0.01	Δ = 0.1	Δ = 0.01	Δ = 0.1
PDRO	1	1.01445	1.15449	1.02483	1.27772	1.04081	1.49383
DLGM	1	1.01708	1.18760	1.02954	1.34303	1.04081	1.49383
THSH	1	1.01067	1.11042	1.02117	1.22807	1.04081	1.49383
DDPK	1.13701	1.17088	1.43984	1.18341	1.65503	1.18341	1.69849
TMVD	1	1.01531	1.16218	1.02621	1.29655	1.04081	1.49383
DAML	1.04632	1.07090	1.28428	1.08377	1.45991	1.08902	1.56301
DFRB	1.18602	1.21503	1.47523	1.22946	1.68862	1.23442	1.77170
DKSR	1	1.01369	1.14431	1.02542	1.28403	1.04081	1.49383
DARO	1	1.01336	1.13604	1.02490	1.27109	1.04081	1.49383
DABO	1.08087	1.10754	1.32544	1.11996	1.49913	1.12498	1.61463
DRZK	1	1.01309	1.14668	1.02621	1.30696	1.04081	1.49383
DOSE	1.20521	1.24204	1.62108	1.25242	1.76833	1.25440	1.80038
PKSH	1	1.02764	1.31573	1.03665	1.43811	1.04081	1.49383
IRDR	1	1.02200	1.24662	1.03535	1.42127	1.04081	1.49383
DALZ	1.11598	1.15232	1.40729	1.15837	1.58136	1.16152	1.66708
DSBH	1	1.01411	1.15603	1.02590	1.29488	1.04081	1.49383
DPAK	1.03365	1.06404	1.37774	1.07481	1.52678	1.07583	1.54409
DJBR	1	1.01527	1.16397	1.02584	1.29450	1.04081	1.49383
KIMI	1	1.01429	1.15097	1.02804	1.32013	1.04081	1.49383
EXIR	1	1.01618	1.17815	1.02826	1.32631	1.04081	1.49383
DSIN	1.02175	1.05252	1.34622	1.06111	1.46680	1.06527	1.52851
ROZD	1	1.02075	1.22630	1.03024	1.35174	1.04081	1.49383
AMIN	1	1.01213	1.12687	1.02297	1.25352	1.04081	1.49383
DZAH	1	1.01924	1.21128	1.02979	1.34710	1.04081	1.49383
ABDI	1	1.01940	1.20921	1.03147	1.36876	1.04081	1.49383
ALBZ	1	1.02474	1.27654	1.03386	1.40068	1.04081	1.49383
DSOB	1	1.02428	1.27051	1.03512	1.41841	1.04081	1.49383

**Table 13:** The results of robust BCC-IO model with different Γ and Δ.

Stocks	BCC-IO	Robust BCC-IO					
		Γ_i_ = 25%		Γ_i_ = 50%		Γ_i_ = 100%	
		Δ = 0.01	Δ = 0.1	Δ = 0.01	Δ = 0.1	Δ = 0.01	Δ = 0.1
PDRO	1	0.98958	0.89733	0.97989	0.81197	0.97545	0.78180
DLGM	1	0.99010	0.90164	0.98300	0.83906	0.98020	0.81818
THSH	1	0.99302	0.93171	0.98607	0.86667	0.98020	0.81818
DDPK	0.98315	0.96800	0.84515	0.96404	0.80717	0.96368	0.80439
TMVD	1	0.99302	0.93171	0.98607	0.86667	0.98020	0.81818
DAML	0.99785	0.98513	0.87741	0.97908	0.82449	0.97769	0.81366
DFRB	0.93931	0.92689	0.82344	0.92119	0.77744	0.91991	0.76595
DKSR	1	0.99123	0.91389	0.98345	0.84432	0.98020	0.81818
DARO	1	0.99302	0.93171	0.98607	0.86667	0.98020	0.81818
DABO	0.98148	0.96630	0.85067	0.95993	0.79770	0.95779	0.78631
DRZK	1	0.99118	0.91132	0.98399	0.84721	0.97998	0.81650
DOSE	0.97150	0.95400	0.81951	0.95173	0.79494	0.95154	0.79244
PKSH	1	0.98738	0.87715	0.98160	0.82909	0.98020	0.81818
IRDR	1	0.98995	0.90031	0.98302	0.84028	0.98020	0.81818
DALZ	0.90532	0.89184	0.80093	0.88531	0.74286	0.88286	0.72334
DSBH	1	0.98904	0.89188	0.98151	0.82743	0.97871	0.80674
DPAK	1	0.98455	0.85267	0.98035	0.81931	0.98020	0.81818
DJBR	1	0.99222	0.92202	0.98463	0.85360	0.98013	0.81769
KIMI	1	0.98784	0.88164	0.98219	0.83380	0.98020	0.81818
EXIR	1	0.98721	0.87557	0.98003	0.81559	0.97816	0.80254
DSIN	1	0.98956	0.89642	0.98413	0.84945	0.98020	0.81818
ROZD	1	0.99302	0.93171	0.98607	0.86667	0.98020	0.81818
AMIN	1	0.99233	0.92465	0.98486	0.85566	0.98020	0.81818
DZAH	1	0.98678	0.87175	0.98171	0.82999	0.98020	0.81818
ABDI	1	0.98907	0.89265	0.98157	0.82831	0.98020	0.81818
ALBZ	1	0.98870	0.88984	0.98185	0.83086	0.98020	0.81818
DSOB	1	0.98284	0.84880	0.97539	0.79472	0.97362	0.77699

**Table 14:** The results of robust BCC-OO model with different Γ and Δ.

Stocks	BCC-OO	Robust BCC-OO					
		Γ_i_ = 25%		Γ_i_ = 50%		Γ_i_ = 100%	
		Δ = 0.01	Δ = 0.1	Δ = 0.01	Δ = 0.1	Δ = 0.01	Δ = 0.1
PDRO	1	1.01205	1.12607	1.01765	1.18954	1.02020	1.22222
DLGM	1	1.01687	1.18696	1.02476	1.28242	1.02498	1.28516
THSH	1	1.00891	1.09266	1.01622	1.17225	1.02041	1.22495
DDPK	1.13393	1.16983	1.41073	1.18154	1.55598	1.18154	1.58623
TMVD	1	1.01489	1.15579	1.02022	1.22239	1.02022	1.22239
DAML	1.03937	1.07008	1.27203	1.07838	1.38154	1.07975	1.40154
DFRB	1.17676	1.20597	1.44294	1.21709	1.48118	1.22023	1.48933
DKSR	1	1.01122	1.11631	1.01774	1.19093	1.02020	1.22222
DARO	1	1.01124	1.11316	1.01760	1.18905	1.02032	1.22383
DABO	1.05556	1.09348	1.31751	1.10704	1.45387	1.10929	1.51272
DRZK	1	1.01246	1.13709	1.02217	1.24885	1.02460	1.28019
DOSE	1.20196	1.23488	1.47071	1.23872	1.49600	1.23928	1.50002
PKSH	1	1.01951	1.21315	1.02012	1.22116	1.02020	1.22222
IRDR	1	1.02200	1.24656	1.03356	1.39814	1.03505	1.41682
DALZ	1.03545	1.05532	1.24298	1.05659	1.26261	1.05670	1.26964
DSBH	1	1.01069	1.11213	1.01737	1.18663	1.02066	1.22824
DPAK	1	1.03567	1.35882	1.04948	1.45341	1.05001	1.46319
DJBR	1	1.01446	1.15041	1.01914	1.20840	1.02020	1.22222
KIMI	1	1.01050	1.10822	1.01655	1.17570	1.02020	1.22222
EXIR	1	1.01299	1.13524	1.01842	1.19915	1.02020	1.22222
DSIN	1	1.03008	1.34081	1.04021	1.41352	1.04600	1.41545
ROZD	1	1.01957	1.21293	1.02125	1.23598	1.02153	1.23976
AMIN	1	1.01038	1.10877	1.01698	1.18134	1.02054	1.22666
DZAH	1	1.01453	1.15428	1.01934	1.21114	1.02076	1.22956
ABDI	1	1.01919	1.20698	1.02531	1.28964	1.02576	1.29550
ALBZ	1	1.01950	1.21321	1.02001	1.21964	1.02020	1.22222
DSOB	1	1.01847	1.20052	1.01967	1.21529	1.02020	1.22222

**Table 15:** The results of robust ADD-CRS model with different Γ and Δ.

Stocks	AD-CRS	Robust AD-CRS					
		Γ_i_ = 25%		Γ_i_ = 50%		Γ_i_ = 100%	
		Δ = 0.01	Δ = 0.1	Δ = 0.01	Δ = 0.1	Δ = 0.01	Δ = 0.1
PDRO	0	3.65643	37.45114	6.05471	64.97821	8.43777	92.81550
DLGM	0	2.91139	30.80281	5.18180	55.73806	6.18585	68.04430
THSH	0	0.69140	7.00529	1.03995	11.13443	1.21859	13.40444
DDPK	30.37334	35.04552	76.31671	36.50649	97.86207	36.73282	99.82867
TMVD	0	3.06794	31.29587	5.10759	54.46824	5.57546	61.33001
DAML	7.50025	12.59990	53.22080	14.09519	76.00912	14.27135	82.05741
DFRB	35.02655	41.24548	90.43678	43.40321	123.41540	43.46187	128.14580
DKSR	0	3.98785	39.96642	6.67440	69.99896	9.10942	100.20360
DARO	0	3.79401	33.93707	5.54876	54.59318	7.45697	82.02667
DABO	18.92588	22.84836	59.42628	25.48741	88.22402	27.05158	103.92240
DRZK	0	2.93318	31.72120	5.35655	59.09796	6.50460	71.55058
DOSE	40.44215	47.40927	116.13680	49.45541	139.73530	49.63439	139.82360
PKSH	0	5.87426	61.33857	7.56819	83.10288	7.75171	85.26880
IRDR	0	4.54350	47.53508	6.88257	75.21511	7.45034	81.95375
DALZ	23.19415	30.46042	85.64939	31.71215	112.53550	31.96632	119.92000
DSBH	0	3.55396	38.64280	6.45874	69.96103	7.63826	83.92442
DPAK	6.18014	11.67245	64.82339	13.60567	87.68671	13.78958	89.94580
DJBR	0	3.34420	34.90031	5.65924	60.31189	7.47842	82.23933
KIMI	0	3.90669	40.29165	6.77174	74.61919	7.25317	79.78490
EXIR	0	4.10206	43.55984	6.61524	72.50218	8.26636	90.93000
DSIN	3.90420	9.43575	60.36415	11.00143	78.67761	11.63905	85.62784
ROZD	0	2.36523	25.84014	3.25817	35.80736	3.26715	35.93865
AMIN	0	3.30466	33.21742	5.30152	55.77706	6.82785	75.10635
DZAH	0	5.30572	55.36421	7.90774	86.84616	8.10041	89.10451
ABDI	0	3.50297	36.16572	5.36715	58.57706	6.15982	67.75799
ALBZ	0	5.24289	53.96454	7.59610	83.25427	8.13529	89.48816
DSOB	0	5.55939	58.23972	7.72743	84.57440	8.16920	89.86116

**Table 16:** The results of robust ADD-VRS model with different Γ and Δ.

Stocks	AD-VRS	Robust AD-VRS					
		Γ_i_ = 25%		Γ_i_ = 50%		Γ_i_ = 100%	
		Δ = 0.01	Δ = 0.1	Δ = 0.01	Δ = 0.1	Δ = 0.01	Δ = 0.1
PDRO	0	3.52118	36.24569	4.58095	45.83396	4.60421	46.05732
DLGM	0	2.83180	30.48616	4.50850	46.91908	4.55274	47.27024
THSH	0	0.67434	6.84956	0.79674	8.35060	0.79674	8.35060
DDPK	29.92194	33.93887	71.97706	34.85604	82.08175	34.88818	82.08175
TMVD	0	2.89362	30.22294	4.57137	45.77568	4.57650	45.90758
DAML	6.63915	12.48890	50.58868	13.95809	64.14033	14.22329	65.10247
DFRB	33.98478	39.38327	79.42991	41.42798	83.88614	41.65799	84.03302
DKSR	0	3.96673	39.74660	4.57449	45.74768	4.58977	45.89810
DARO	0	3.72513	33.82268	3.82967	38.44936	3.82967	38.44936
DABO	13.88503	22.49131	57.35480	25.19395	76.04247	26.85489	81.11245
DRZK	0	2.78368	29.43598	4.70625	49.82927	5.52904	58.10281
DOSE	39.67503	44.32403	82.95036	44.95094	84.21140	44.95094	84.21651
PKSH	0	4.51065	45.13373	4.58770	45.88366	4.59585	45.96445
IRDR	0	4.53210	47.14990	6.44546	69.94132	6.66532	71.45331
DALZ	7.82084	12.29129	52.09176	12.38475	53.44778	12.38475	53.59838
DSBH	0	3.09388	31.81045	4.98775	50.27810	5.10502	51.71274
DPAK	0	6.65582	63.52113	9.20924	79.83819	9.30589	80.56057
DJBR	0	3.33418	34.35464	4.55439	45.61685	4.58307	45.86061
KIMI	0	3.46690	35.17490	4.75557	47.57243	4.75819	47.59474
EXIR	0	3.64449	37.30933	4.73618	47.52315	4.76861	47.96000
DSIN	0	5.20796	59.53734	7.09164	73.53110	8.01303	73.53110
ROZD	0	2.24241	25.23877	3.23651	34.11476	3.23651	34.11476
AMIN	0	3.28046	32.87439	4.56505	46.69973	4.92826	49.53274
DZAH	0	4.61072	46.57645	6.33814	64.52310	6.82637	69.47096
ABDI	0	3.44291	35.06341	4.46196	47.11460	4.53616	47.96477
ALBZ	0	4.43154	44.34075	4.57671	45.77012	4.59096	45.90900
DSOB	0	4.27479	42.95009	4.57773	45.77941	4.59334	45.93082

**Table 17:** The results of Robust Mean-Semi Variance-Liquidity (RMSVL) model with different Γ and Δ.

Stocks & Portfolio		MSVL	Robust MSVL					
			Γ_i_ = 25%		Γ_i_ = 50%		Γ_i_ = 100%	
			Δ = 0.01	Δ = 0.1	Δ = 0.01	Δ = 0.1	Δ = 0.01	Δ = 0.1
Weight of Selected Stocks from Phase .1 in Portfolio	PDRO	0.01000	0.01000	0.07197	0.01000	0.06643	0.01000	0.01000
	DLGM	0.18614	0.18366	0.13747	0.18365	0.06998	0.18526	0.06049
	THSH	0.01739	0.02382	0.01000	0.01948	0.01000	0.01753	0.01000
	TMVD	0.01000	0.01023	0.03986	0.01285	0.06154	0.01000	0.04753
	DKSR	0.01000	0.01000	0.11040	0.01000	0.05664	0.01000	0.13109
	DARO	0.13683	0.14357	0.13075	0.13751	0.06655	0.13762	0.07597
	DJBR	0.17334	0.17639	0.13194	0.18078	0.35142	0.18254	0.36135
	KIMI	0.01000	0.01000	0.01576	0.01000	0.01339	0.01000	0.01000
	ROZD	0.03011	0.02286	0.01000	0.02137	0.01000	0.02105	0.01000
	AMIN	0.41618	0.40946	0.34184	0.41436	0.29405	0.41600	0.28356
Risk (SV) of Portfolio		0.00480	0.00496	0.00668	0.00502	0.00802	0.00503	0.00869

**Table 18:** The Results of Robust Mean-Absolute Deviation-Liquidity (RMADL) model with different Γ and Δ.

Stocks & Portfolio		MADL	Robust MADL					
			Γ_i_ = 25%		Γ_i_ = 50%		Γ_i_ = 100%	
			Δ = 0.01	Δ = 0.1	Δ = 0.01	Δ = 0.1	Δ = 0.01	Δ = 0.1
Weight of Selected Stocks from Phase .1 in Portfolio	PDRO	0.01000	0.01000	0.01000	0.01000	0.06276	0.01000	0.01000
	DLGM	0.16973	0.17994	0.09240	0.17527	0.06367	0.17443	0.01000
	THSH	0.01000	0.01000	0.01000	0.01000	0.01000	0.01000	0.01000
	TMVD	0.09270	0.08418	0.06303	0.07989	0.05814	0.07870	0.01000
	DKSR	0.01000	0.01000	0.13372	0.01000	0.05309	0.01000	0.01000
	DARO	0.27319	0.27135	0.15836	0.27842	0.07215	0.27970	0.22800
	DJBR	0.01000	0.01413	0.13917	0.02171	0.36385	0.02352	0.46564
	KIMI	0.01000	0.01000	0.01000	0.01000	0.01000	0.01000	0.01000
	ROZD	0.01980	0.01000	0.01000	0.01000	0.01000	0.01000	0.01000
	AMIN	0.39457	0.40039	0.37332	0.39472	0.29635	0.39366	0.23635
Risk (AD) of Portfolio		0.08748	0.09020	0.11894	0.09089	0.13527	0.09109	0.13816

As can be seen in Tables 11–18 and Figs 4–11, the results indicate that, as the budget of robustness Γ increases from 0% to 100% for uncertain parameters, the objective function gets worse. Also, as the perturbations Δ increases from 0.01 to 0.1, the objective function gets worse than the nominal problem. It should be noted that the expected return and the expected liquidity of portfolio in both of robust MSVL and robust MADL models are set equal to 0.013 and 14.50, respectively.

In the end of this section, the portfolio performance based on RMSVL and RMADL models will be analyzed. Accordingly, five popular measures including excess mean return (EMR), downside deviation (DD), Sharpe ratio (SHR), information ratio (IR), and Sortino ratio (SOR) are applied. A brief description of these measures is introduced as follows:

**EMR**: Describe portfolio’s reward over market index or the difference between portfolio return and market index return. EMR is calculated by Eq (32), where *R*_*P*_ and *R*_*I*_ denote on portfolio return and market index return, respectively. Please note that higher values of EMR are desirable.

$$EMR=\frac{1}{T}\;\sum_{t=1}^{T}\;(R_{P}(t)-R_{I}(t))$$ (32)

**DD**: Describe the underachievement of portfolio from the market index. DD is calculated by Eq (33). Please note that lower values of DD are desirable.

$$DD=\frac{1}{\sqrt{T}}\;\sqrt{\;\sum_{t=1}^{T}\;{\left(Min\;\{\;(R_{P}(t)-R_{I}(t)),0\;)\right)}^{2}\;}$$ (33)

**SHR**: Describe the average earned return over risk-free return rate per unit of standard deviation. SHR is calculated by Eq (34), where *E*(*R*_*P*_), *R*_*f*_, and *σ*(*R*_*P*_) denote on average portfolio return, risk-free return rate, and standard deviation of portfolio return. Please note that higher values of SHR are desirable.

$$SHR=\left\{ \begin{array}{cc} \frac{E(R_{P})-R_{f}}{\sigma (R_{P})} & if\;E(R_{P})>R_{f}\\ 0 & if\;E(R_{P})\leq R_{f} \end{array}\right.$$ (34)

**IR**: Describe the risk-adjusted returns of a financial asset or portfolio relative to a certain benchmark and it is calculated by Eq (35). Please note that higher values of IR are desirable.

$$IR=\left\{ \begin{array}{ll} \frac{EMR}{\sigma (R_{P}-R_{I})} & if\;EMR>0\\ 0 & if\;EMR\leq 0 \end{array}\right.$$ (35)

**SOR**: Describe the return per unit risk and it is calculated by Eq (36). Please note that higher values of SOR are desirable.

$$SOR=\left\{ \begin{array}{ll} \frac{EMR}{DD} & if\;EMR>0\\ 0 & if\;EMR\leq 0 \end{array}\right.$$ (36)

Now, by applying Eqs (32) to (36), all performance measures are calculated for RMSVL and RMADL models. It should be explained that the risk-free return rate is 0.10. The results of EMR, DD, SHR, IR, and SOR are presented in Table 19:

**Table 19:** The results of performance measures for RMSVL and RMADL models.

Proposed Model	EMR	DD	SHR	IR	SOR
RMSVL	0.0602019	0.0703496	0.4271998	0.3619597	0.8557527
RMADL	0.0601969	0.0763361	0.4267645	0.3512621	0.7885770

According to the results, it is obviously observed that both of two proposed models including RMSVL and RMADL are effective to construction of optimal portfolio. In other words, the proposed approach is capable to achieve desirable return in comparison with risk-free return rate. It should be noted that the performance of RMSVL model is marginally better than RMADL model under all five measures.

## 7. Conclusions and future research directions

In this study, a novel approach for the portfolio construction problem is proposed in order to deal with data uncertainty, increasing conservatism levels of the investment process, decreasing computational complexity, and assessing comprehensive of stocks. Accordingly, this study presents six RDEA models based on the most widely cited and popular classic data envelopment analysis models in the first phase and two robust portfolio optimization models including robust mean-semi variance-liquidity and robust mean-absolute deviation-liquidity in the second phase. It is worth mentioning here that the uncertainty is considered on all data in two phases including input and output data in DEA models and financial parameters in MSVL and MADL models by robust optimization approach. Finally, a real-life case study from the Tehran stock exchange is implemented to demonstrate the applicability of the proposed two-phase robust portfolio selection and optimization approach and exhibit the efficacy and effectiveness of the presented method in this paper. Additionally, the sensitivity analysis of all robust models of this study is illustrated. The results show that the proposed approach is effective for portfolio construction under uncertainty environment. Also, the computational complexity for consideration cardinality constraint in portfolio optimization models by applying the presented two phases approach is decreased. In other words, this approach does not need any meta-heuristic algorithm for solving the portfolio optimization model with investment constraint. In the end, the main contributions of this study can be summarized as follows:

- The paper introduces a novel two-phase portfolio selection and optimization approach.
- Six RDEA models are proposed in order to stock performance measurement under uncertainty.
- Two robust portfolio optimization models with different risk measures are presented.
- Sensitivity analysis of all eight robust models in this study are illustrated.
- The proposed approach is implemented in a real- life case study of Tehran stock exchange.

For future studies, uncertainty programming approaches such as fuzzy mathematical programming and chance-constrained programming can be applied in order to deal with another type of data uncertainty [115–119]. Moreover, data-driven robust optimization (DDRO) approach can be employed for proposing data-driven robust portfolio optimization (DDRPO) models [120–123].

## Supporting information

S1 Data (DOCX)
