# Supplementary material for: A novel two-phase robust portfolio selection and optimization approach under uncertainty: A case study of Tehran stock exchange
Source: PLoS One. 2020 Oct 12;15(10):e0239810. doi: 10.1371/journal.pone.0239810 (PMC7549800; doi:10.1371/journal.pone.0239810)
Supplement: S1 Data — (DOCX) [file pone.0239810.s001.docx]

**Real-World Data from Tehran Stock Exchange - Pharmaceutical Industry (March 2013-March 2014)**

| Stocks |  | Inputs | | | | |  | Outputs | | | |
| --- | --- | --- | --- | --- | --- | --- | --- | --- | --- | --- | --- |
|  |  | I (1) | I (2) | I (3) | I (4) | I (5) |  | O (1) | O (2) | O (3) | O (4) |
| PDRO |  | 7.43 | 1.18 | 1.22 | 1.03 | 0.02 |  | 3344 | 1.93 | 157.67 | 59.33 |
| DLGM |  | 13.38 | 0.49 | 3.87 | 0.70 | 0.03 |  | 213 | 2.06 | 183.48 | 133.33 |
| THSH |  | 11.58 | 0.59 | 2.85 | 0.01 | 0.02 |  | 799 | 0.69 | 110.28 | 30.16 |
| DDPK |  | 7.70 | 0.86 | 2.27 | 0.54 | 0.05 |  | 693 | 2.73 | 122.76 | 56.85 |
| TMVD |  | 6.58 | 1.16 | 1.00 | 0.64 | 0.02 |  | 2965 | 1.04 | 166.99 | 10.66 |
| DAML |  | 8.70 | 0.87 | 3.91 | 0.57 | 0.03 |  | 1386 | 1.98 | 156.08 | 2.74 |
| DFRB |  | 7.76 | 1.07 | 1.84 | 1.40 | 0.03 |  | 1277 | 2.04 | 164.07 | 31.17 |
| DKSR |  | 8.96 | 0.97 | 1.36 | 1.48 | 0.03 |  | 121 | 2.64 | 228.88 | 369.42 |
| DARO |  | 7.93 | 7.07 | 0.10 | 1.27 | 0.03 |  | 1553 | 1.85 | 187.63 | 54.67 |
| DABO |  | 9.03 | 0.86 | 3.44 | 0.71 | 0.03 |  | 1357 | 2.30 | 143.68 | 93.15 |
| DRZK |  | 7.91 | 0.96 | 1.72 | 0.68 | 0.03 |  | 1493 | 2.88 | 167.43 | 96.65 |
| DOSE |  | 18.43 | 1.06 | 1.23 | 1.56 | 0.04 |  | 997 | 1.92 | 169.70 | 67.00 |
| PKSH |  | 6.41 | 0.90 | 5.95 | 1.67 | 0.03 |  | 528 | 0.73 | 227.86 | 53.22 |
| IRDR |  | 7.47 | 0.72 | 3.00 | 1.09 | 0.03 |  | 306 | 1.59 | 187.99 | 230.39 |
| DALZ |  | 7.46 | 1.28 | 1.21 | 1.49 | 0.03 |  | 956 | 2.49 | 205.22 | 111.30 |
| DSBH |  | 8.39 | 1.35 | 0.86 | 1.60 | 0.04 |  | 2340 | 2.91 | 155.82 | 95.56 |
| DPAK |  | 6.82 | 0.79 | 4.43 | 1.30 | 0.05 |  | 666 | 2.52 | 177.08 | 119.82 |
| DJBR |  | 6.94 | 1.21 | 0.94 | 0.94 | 0.03 |  | 659 | 3.14 | 219.36 | 122.76 |
| KIMI |  | 6.81 | 0.73 | 2.28 | 6.24 | 0.21 |  | 227 | 5.74 | 147.27 | 438.33 |
| EXIR |  | 8.20 | 0.82 | 5.16 | 1.14 | 0.03 |  | 1283 | 3.14 | 198.36 | 118.24 |
| DSIN |  | 7.52 | 1.21 | 0.84 | 0.97 | 0.03 |  | 1222 | 1.80 | 174.39 | 94.68 |
| ROZD |  | 8.84 | 1.01 | 0.95 | 0.28 | 0.07 |  | 131 | 1.46 | 26.37 | 286.26 |
| AMIN |  | 5.73 | 0.97 | 1.45 | 0.74 | 0.04 |  | 696 | 4.15 | 163.71 | 230.03 |
| DZAH |  | 5.40 | 0.95 | 2.83 | 1.20 | 0.07 |  | 2699 | 2.35 | 44.51 | 129.27 |
| ABDI |  | 10.22 | 0.60 | 4.81 | 0.59 | 0.03 |  | 404 | 2.21 | 181.41 | 83.42 |
| ALBZ |  | 6.90 | 1.00 | 1.93 | 1.41 | 0.03 |  | 418 | 1.49 | 228.42 | 104.07 |
| DSOB |  | 6.75 | 1.06 | 1.57 | 1.46 | 0.03 |  | 655 | 2.65 | 221.73 | 104.58 |
| Mean |  | 8.34 | 1.18 | 2.33 | 1.21 | 0.04 |  | 1088.44 | 2.31 | 167.34 | 123.22 |
| SD |  | 2.58 | 1.17 | 1.50 | 1.07 | 0.04 |  | 851.51 | 1.03 | 48.01 | 101.97 |
| Max |  | 18.43 | 7.07 | 5.95 | 6.24 | 0.21 |  | 3344 | 5.74 | 228.88 | 438.33 |
| Min |  | 5.40 | 0.49 | 0.10 | 0.01 | 0.02 |  | 121 | 0.69 | 26.37 | 2.74 |

|  | Financial Criteria |  | Symbol |  | Description |
| --- | --- | --- | --- | --- | --- |
| Inputs | Price to Earnings Ratio (P/E) |  | I (1) |  | Stock price divided by net income per share |
|  | Quick Ratio |  | I (2) |  | Total current assets minus inventory divided by total current liabilities |
|  | Solvency Ratio-II |  | I (3) |  | Total liability divided by shareholders equity |
|  | Beta (*β*) |  | I (4) |  | Systematic Risk |
|  | Standard Deviation (*σ*) |  | I (5) |  | Non-Systematic Risk |
| Outputs | Earnings per Share (EPS) |  | O (1) |  | Net income minus dividends divided by common shares |
|  | Rate of Return |  | O (2) |  | Proportion of gain or loss on an investment over a specified period |
|  | Rate of Liquidity |  | O (3) |  | Degree which presents stock ability to be bought or sold in the market quickly |
|  | Earnings per Share Growth Rate |  | O (4) |  | Current quarters EPS divided by the previous quarters EPS minus one |

**The Monthly Rate of Return of Stocks - Pharmaceutical Industry (March 2013-March 2014)**

| Periods | Selected Stocks in Phase 1 | | | | | | | | | |
| --- | --- | --- | --- | --- | --- | --- | --- | --- | --- | --- |
|  | PDRO | DLGM | THSH | TMVD | DKSR | DARO | DJBR | KIMI | ROZD | AMIN |
| Period 1^th^ | 0.0160 | 0.2266 | -0.0031 | 0.1359 | 0.1685 | 0.0097 | 0.0508 | 0.2122 | 0.0071 | 0.1094 |
| Period 2^th^ | -0.1193 | -0.0638 | -0.2154 | 0.0289 | 0.0012 | 0.0563 | 0.1100 | 0.5835 | 0.0049 | 0.1238 |
| Period 3^th^ | 0.3156 | 0.1300 | 0.1015 | 0.3195 | 0.3108 | 0.0468 | 0.3073 | 0.6966 | 0.0497 | 0.1197 |
| Period 4^th^ | 0.2472 | 0.4190 | 0.1030 | 0.1672 | 0.5002 | 0.7275 | 0.5629 | 0.5675 | 0.0829 | 0.0322 |
| Period 5^th^ | 0.1063 | 0.1474 | 0.0232 | -0.1552 | -0.0924 | 0.0633 | -0.0493 | -0.2953 | 0.0188 | 0.1704 |
| Period 6^th^ | 0.0909 | -0.1696 | 0.1883 | 0.0055 | 0.0133 | 0.1084 | 0.0361 | -0.0666 | 0.0028 | 0.3193 |
| Period 7^th^ | 0.2347 | 0.1540 | 0.0636 | 0.1367 | 0.3673 | 0.0068 | 0.1900 | -0.0393 | 0.0118 | 0.3822 |
| Period 8^th^ | 0.3047 | 0.3167 | 0.0343 | 0.1907 | 0.3148 | 0.0769 | 0.4905 | 1.2232 | 0.0005 | 0.5867 |
| Period 9^th^ | 0.1981 | 0.1549 | 0.0480 | 0.0689 | 0.2201 | 0.2104 | 0.2029 | 0.5297 | 0.1466 | 0.1842 |
| Period 10^th^ | -0.1011 | 0.0860 | 0.0240 | -0.0097 | 0.0317 | 0.2085 | -0.1096 | -0.0594 | 0.1328 | -0.0580 |
| Period 11^th^ | 0.0028 | -0.0609 | 0.2036 | -0.0174 | -0.2285 | -0.1966 | -0.1507 | -0.1246 | 0.0161 | 0.0803 |
| Period 12^th^ | 0.0483 | -0.0157 | 0.0332 | -0.0210 | 0.0523 | 0.0066 | 0.2050 | -0.0113 | 0.5774 | -0.0563 |
| Average Return | 0.1120 | 0.1104 | 0.0504 | 0.0708 | 0.1383 | 0.1104 | 0.1538 | 0.2680 | 0.0876 | 0.1662 |

**The Monthly Rate of Liquidity of Stocks - Pharmaceutical Industry (March 2013-March 2014)**

| Periods | Selected Stocks in Phase 1 | | | | | | | | | |
| --- | --- | --- | --- | --- | --- | --- | --- | --- | --- | --- |
|  | PDRO | DLGM | THSH | TMVD | DKSR | DARO | DJBR | KIMI | ROZD | AMIN |
| Period 1^th^ | 9.83 | 14.76 | 3.91 | 8.41 | 14.2 | 6.62 | 12.19 | 14.07 | 1.20 | 10.67 |
| Period 2^th^ | 18.08 | 11.97 | 14.55 | 16.38 | 21.04 | 19.55 | 19.43 | 5.31 | 1.33 | 14.79 |
| Period 3^th^ | 17.78 | 1.81 | 15.79 | 14.24 | 18.37 | 8.69 | 12.33 | 7.35 | 3.51 | 7.01 |
| Period 4^th^ | 10.52 | 14.66 | 16.47 | 20.09 | 18.36 | 15.84 | 20.57 | 11.23 | 3.34 | 4.62 |
| Period 5^th^ | 15.96 | 15.00 | 9.43 | 18.88 | 18.54 | 18.10 | 19.31 | 19.15 | 3.41 | 5.21 |
| Period 6^th^ | 18.77 | 14.35 | 12.73 | 18.55 | 19.72 | 14.76 | 19.79 | 20.07 | 1.00 | 17.48 |
| Period 7^th^ | 19.10 | 18.94 | 17.66 | 19.03 | 20.52 | 18.52 | 19.59 | 5.76 | 1.20 | 9.58 |
| Period 8^th^ | 15.99 | 18.60 | 9.09 | 18.89 | 19.69 | 17.33 | 19.59 | 4.97 | 0.67 | 12.88 |
| Period 9^th^ | 20.10 | 19.80 | 9.34 | 20.74 | 20.78 | 14.80 | 20.65 | 13.87 | 0.97 | 20.47 |
| Period 10^th^ | 17.74 | 15.31 | 6.29 | 18.26 | 18.77 | 18.05 | 14.78 | 18.8 | 1.55 | 18.20 |
| Period 11^th^ | 13.63 | 18.84 | 12.82 | 15.49 | 17.25 | 20.07 | 20.31 | 14.31 | 4.03 | 17.05 |
| Period 12^th^ | 14.23 | 18.00 | 8.44 | 17.98 | 19.39 | 19.11 | 19.25 | 16.73 | 2.70 | 18.92 |
| Average Liquidity | 15.98 | 15.17 | 11.38 | 17.25 | 18.89 | 15.95 | 18.15 | 12.64 | 2.08 | 13.07 |
